# KDELR1 regulates chondrosarcoma drug resistance and malignant behavior through Intergrin-Hippo-YAP1 axis

**DOI:** 10.1038/s41419-024-07264-7

**Published:** 2024-12-23

**Authors:** Huabin Yin, Dongjie Jiang, Yongai Li, Wenjun Chen, Jie Zhang, Xinghai Yang, Jinbo Hu, Haifeng Wei

**Affiliations:** 1 Department of Orthopedics, Shanghai General Hospital, Shanghai Jiao Tong University School of Medicine, No.85 Wujin Road, Hongkou District, Shanghai, 200080 China; 2 Spinal Tumor Center, Department of Orthopaedic Oncology, No.905 Hospital of PLA Navy, Changzheng Hospital, Naval Medical University, No.415 Fengyang Road, Shanghai, 200003 China

**Keywords:** Bone cancer, Bone cancer, Prognostic markers

## Abstract

Chondrosarcoma (CS) is the second most common primary bone malignancy, known for its unique transcriptional landscape that renders most CS subtypes resistant to chemotherapy, including neoadjuvant chemotherapy commonly used in osteosarcoma (OS) treatment. Understanding the transcriptional landscape of CS and the mechanisms by which key genes contribute to chemotherapy resistance could be a crucial step in overcoming this challenge. To address this, we developed a single-cell transcriptional map of CS, comparing it with OS and normal cancellous bone. Our analysis revealed a specific increase in KDEL receptor 1 (KDELR1) expression in CS, which was closely associated with CS prognosis, tumor aggressiveness, and drug resistance. KDELR1 plays a key role in regulating membrane protein processing and secretion, as well as contributing to tumor extracellular matrix (ECM) formation and drug resistance. Further investigation using mass spectrometry proteomics and transcriptomics uncovered KDELR1’s involvement in modulating the Hippo-YAP pathway activity in CS cells. The KDELR1-Integrin-PLCγ-YAP1 axis emerges as a critical process mediating drug resistance and malignant behavior in CS, offering novel insights and potential therapeutic targets for CS treatment.

## Introduction

Chondrosarcomas (CSs) are the second most frequent primary bone cancers after osteosarcomas (OSs) [1]. The most frequently encountered CS subtypes are grade1/2/3 (85–90%), which are called conventional CSs based on histopathology [2, 3]. Nonconventional CS variants include clear cell, mesenchymal, and dedifferentiated CS; mesenchymal and dedifferentiated CS are highly malignant subtypes with poor 5-year survival rates (51% and 18% respectively) [4, 5]. Several researchers found that patients with dedifferentiated and mesenchymal CS who received chemotherapy had a reduced risk of recurrence and death. However, most CS subtypes are considered refractory to chemotherapy. Unfortunately, very little is known about the exact causes and mechanisms of CS drug resistance. As a consequence, neoadjuvant chemotherapy is generally not effective for the poor response of CS cells to Cisplatin (causing DNA damage), Dasatinib (targeting Bcr-Abl and Src), and Pazopanib (inhibiting angiogenic activity) [6–10]. It greatly restricts the excisional surgery, which is the main treatment for most types of CS. Therefore, novel therapeutic targets of CS are urgently needed.

The Hippo signaling pathway is one of the critical pathways in embryonic development. As the downstream effector of Hippo signaling, YAP1 involves in chondrogenesis and chondrocyte differentiation during normal development [11]. From the point of the pathology, the redundant translocation of dephosphorylated YAP1 from the cytoplasm to the nucleus was a bad prognostic factor in CS [12, 13]. In CS-related researches, YAP ablation repressed the malignant behaviors of CS, including tumorsphere formation, proliferation, invasion, migration, and progression in vitro [14, 15]. CS cells possess the ability of producing the cartilage extracellular matrix (ECM), including several collagen subtypes and integrins with different pairing assembling of α and β-subunits, characterizing the ECM and microenvironment of CS, providing external environmental supporting for CS cells and mediating the cell-cell interactions [16]. Enhancement of Hippo-YAP signaling pathway reflects cell adhesion and mechanical signals in ECM [17]. Simultaneously, the ON/OFF switch of Hippo-YAP signaling pathway is regulated by mechanical strains and modifications of surrounding ECM composition [18]. However, very little is known about the regulation between ECM of CS and the ON/OFF switch of Hippo-YAP signaling pathway.

Recently, inter-organelle crosstalk between the endoplasmic reticulum (ER) and Golgi was proved a vital factor for tumor cells death and ER-Golgi trafficking was considered as a new target for anti-cancer therapy [19]. The precise and fluent execution process of ER-Golgi trafficking is hinged on KDEL receptors (KDELRs), which are in ER, Golgi, and ER-Golgi intermediate compartment (ERGIC). Concretely, KDEL receptors located its N-terminal region to the lumen of ER, Golgi and ERGIC, and the C-terminus to cytoplasm [20, 21], which endows KDELRs with retrieving the HDEL-containing protein from Golgi to endoplasmic reticulum after recognizing and binding these proteins. Intriguingly, the progression and tumorigenesis of CSs are related to the function of both of ER [22–24] and Golgi [25, 26], resulting the expression alteration of CS-related secretory or membrane proteins, and subsequently transducing survival signaling pathways and maintaining the ECM homeostasis. As the effector of ER-Golgi trafficking, whether KDELR regulates Hippo YAP by affecting cartilage ECM needs further investigation.

In our study, we revealed the crucial role of KDELR1 in CS progression, proliferation, migration, and drug-sensitivity by enhancing the Hippo-YAP1 signaling pathway via ITG-α3/5β5-PLCγ-MAP4K4 axis. KDELR1 is a dependable biomarker of CS malignancy degree and prognosis and a novel and promising therapeutic target for CS.

## Material and methods

### Human samples

A total of 110 samples including 29 cases of osteochondroma specimens and 81 cases of CS specimens (28 cases of Grade I, 28 cases of Grade II, 25 cases of Grade III) were obtained from patients who were diagnosed as having CS and received surgical treatment at Shanghai General Hospital of Shanghai Jiao Tong University (Shanghai, China) between 2010 and 2022.The Institutional Review Board of Shanghai General Hospital approved these research studies (2019SQ031), and informed consent was obtained.

### Cell lines and primary cultures

HEK293T cells, human normal cartilage cell line CHON-001, and CS cell lines SW1353 and Hs 819.T cells were cultured in DMEM or DMEM/F12 medium supplemented with 10% fetal bovine serum, penicillin and streptomycin.

### RNA interference to knock down gene expression

Stable cell lines were utilized for protein profiling, sequencing, and animal experiments, while cell lines that were transiently transfected with siRNA were employed for other experiments. The cDNA clone of KDELR1 and YAP1 were purchased from OriGene (Rockville, MD, USA). The coding sequence of KDELR1 and YAP1 were amplified by PCR and inserted downstream of promoter in the pcDNA3.1 vector. siRNAs specific for KDELR1 and ctrl siRNA were purchased from Tsingke Biotech (Beijing, China). Transient transfection was performed using Lipofectamine 3000 Transfection Reagen (Invitrogen, L3000015). KDELR1-KD CS cells were stable cell line and were generated with shRNA by the lentiviral transduction. Concretely, 3 μg of psPAX2, 3 μg of pMD2G, and 3 μg of recombinant plasmid were added to 250 μl DMEM, and then mixed with another 250 μl DMEM containing 18 ul lipo3000 and 18 ul P3000. After mixing well and incubating at room temperature for 15 min, the mixture was added to a 60 mm dish of HEK293T cells with a 60-70% confluency. After 24 h and 48 h, lentivirus-containing supernatant was collected and floating cells were removed by 0.45 μm filters. And then, a Lentivirus Concentration Kit (Genomeditech, GM-040801) was used to obtain the lentivirus precipitation, which would be dissolved in 200 μl basal medium. When CS cells reached a 50-60% confluency, the 200 μl medium containing lentivirus were added into complete medium (without penicillin and streptomycin) to infect CS cells with polybrene for 48 h. Then, antibiotic selection was implemented for killing non-infected cells. And the polyclonal cell populations were the stable cell line. The overexpressed lentivirus of active-YAP1 and control lentivirus (OBiO Technology Corp., Ltd, Shanghai, China) was used to infect CS cells to get stably cells through puromycin selection.

### Western blot analysis

The protein in normal cartilage tissue and CS samples was immersed in RIPA buffer with PMSF and then extracted by stainless-steel grinding balls and tissue homogenizer. The protein from cell line or human samples was lysed in RIPA buffer or SDS-loading buffer. We ran the gel for one-two hours at 120 V and transferred the protein from the gel to the nitrocellulose membrane. After blocking the membrane for one hour at room temperature using 5% non-fat milk, we incubated it overnight at 4°C with recommended dilutions of primary antibodies (Table S1). After washing, we incubated the membrane with HRP-conjugated secondary antibody (Beyotime A0216 or A0208) in PBST.

### Transwell

Complete medium 500 μl per well was added to 24-well plate. Transwell inserts were utilized, 50,000 cells in 150 ul complete medium (2% fetal bovine serum) were seeded into top chamber. After cultured in incubator for 24 h for SW1353 and Hs 819.T, cells on the bottom of membrane were fixed with 4% paraformaldehyde solution and permeated with 0.1% triton X-100. Then, 0.1% crystal violet was employed to stain CS cells.

### Cell counting Kit-8 test

CS cells were prepared and seeded in a 96-well plate with a concentration of 2000 cells per well. Cisplatin was added with a dose gradient from 0 to 100 uM. 10 ul CCK8 (MCE, HY-K0301) was mixed with 100 μl complete medium. After one hour incubation the OD-450 values were read using microplate reader (Thermo Fisher Scientific).

### LC-MS analysis and RNA sequencing

Total RNA was extracted by Trizol (Thermofisher, 15596018). Its concentration and purity were determined by NanoDrop ND-1000 (NanoDrop, Wilmington, DE, USA). And RNA integrity was tested by Bioanalyzer 2100 (Agilent, CA, USA). Oligo (dT) magnetic beads (Dynabeads Oligo (dT), cat.25-61005, Thermo Fisher, USA) were used to enrich the mRNA with a poly A tail. Then, a NEBNext Magnesium RNA Fragmentation Module (E6150S) was used to fragment RNA into small pieces by divalent cations under elevated temperature, and a cDNA library was constructed using Invitrogen SuperScriptTM II Reverse Transcriptase (1896649). Finally, we performed paired-end sequencing using Illumina NovaseqTM 6000 according to standard procedures, with the sequencing mode of PE150. Genes differential expression analysis was performed by DESeq2 software between two different groups (and by edgeR between two samples). The genes with the parameter of false discovery rate (FDR) below 0.05 and absolute fold change ≥ 1 were considered differentially expressed genes (DEGs). DEGs were then subjected to enrichment analysis of GSVA.

As for LC-MS analysis, the two groups of cells were harvested by scrapers on the ice, and frozen by liquid nitrogen for three-four hours. Protein of KDELRI-siRNA cells and siRNA-ctrl cells was extracted by standard sample preparation workflow including reduction, alkylation, acetone precipitation 12 h at -20°C, trypsin digestion. And then, differentially expressed proteins between KDELRI-siRNA cells and siRNA-ctrl cells were determined by mass spectrometry using a tims TOF mass spectrometer (Bruker) of OBiO Technology Corp., Ltd (Shanghai, China). And gene Ontology (GO) analysis was conducted by using the R package, which revealed the functional enrichment of the biology process (BP), cell component (CC), and molecular function (MF) for these differentially expressed proteins between KDELRI-siRNA cells and siRNA-ctrl cells.

### Nuclear and cytoplasm extraction

To avoid proteolysis, scrapers were used to harvest the CS cells. Then, the nuclear and cytoplasmic protein were obtained using the Nuclear and Cytoplasmic Protein Extraction Kit (Meilunbio, MA0211). Protein concentration was determined with a BCA protein assay kit (Thermo Fisher Scientific, 23225). The nuclear and cytoplasmic protein was prepared for western blot.

### Immunohistochemistry (IHC) and immunofluorescence (IF)

Slides were deparaffinized by a sequential immersion of xylene I-xylene II, 100% alcohol, 95% alcohol, 70% alcohol, 50% alcohol, each immersion for 5 min. Then, triton X-100, sodium citrate buffer, and 3% H_2_O_2_ solution in methanol were used for permeation, antigen retrieval and elimination of endogenous peroxidase activity, respectively. The primary antibody of KDELR1 and biotinylated secondary antibody were incubated with slides after blocking nonspecific binding. HRP conjugates and DAB substrate solution were used to reveal the color of antibody staining. After fixation, permeabilization, blocking, primary antibodies of YAP1 were used for IF assay. We separated nuclear staining from the protein of interest staining in IHC images using Image J software [27]. According to the percentage of cells with positive nuclear staining, the sections were scored into six groups: 0, no nuclear staining; 1, < 10% positive cells; 2, 10-25% positive cells; 3, 26-50% positive cells; 4, 51-75% positive cells; and 5, > 75% positive cells. Tumors with a staining score ≥3 were designated as high KDELR1 expression and those with a staining score < 3 as low KDELR1 expression. The statistical significance of different groups was calculated by Two-tailed Student’s t test by GraphPad Prism 8 software. The significance level was set to 0.05.

### In vivo animal studies

Five weeks old BALB/c nude WT mice were purchased from Shanghai SLAC Laboratory Animal Co., Ltd. A total of 35 mice were divided into five groups. CS cells stably transduced with control or KDELR1 shRNA were prepared and injected into subcutaneous flank with 3 ×10^6^ cells per 100 ul, which were mixed with 100 μl of matrigel. For the therapy, once the tumors became palpable, mice injected with WT cells or KDELR-KD cells were randomly divided into two groups, respectively, for the administration of drugs. Cisplatin was prepared with physiological saline at a dose of 4 mg/kg and injected intraperitoneally at a dosage of 0.2 ml/10 g, administered every five days, a total of four times. These in vivo experiments were conducted in the Animal Center of Shanghai General Hospital of Shanghai Jiao Tong University and were performed in accordance with the guidelines of the Ethics Committee of Shanghai General Hospital of Shanghai Jiao Tong University [28].

### Single-cell RNA sequencing (scRNA-seq)

The cell suspension was loaded into Chromium microfluidic chips with 3’ chemistry and barcoded with a 10× Chromium Controller (10X Genomics). Sequencing libraries constructed by the Sequencing Core at Shanghai Xulan Biotechnology Co., LTD, Sequencing was performed with Illumina (HiSeq 2000). All downstream single-cell analyses were performed using Cell Ranger and Seurat.

### Statistics

All data are presented as mean ± SD. The statistical significance of different groups was calculated by GraphPad Prism 8 software. Two-tailed Student’s t test and one-way ANOVA were used for two and three or more groups’ comparisons, respectively. The significance level was set to 0.05.

## Results

### KDELR1 was specifically highly expressed in CS tumor cells

**Fig. 1:**
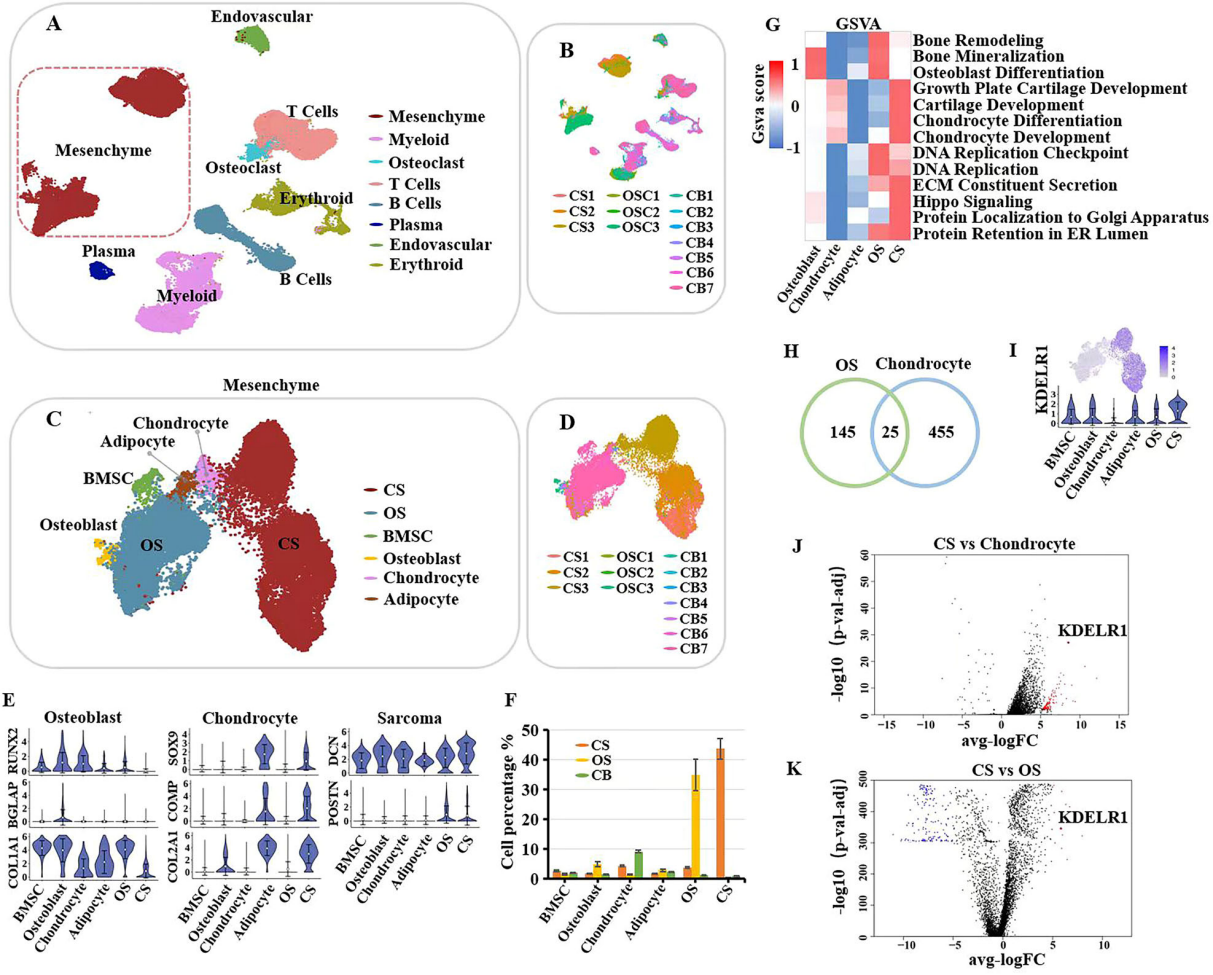
KDELR1 is an oncogenic regulator of CS. **A, B** Uniform Manifold Approximation and Projection (UMAP) plot of indicated genes in merged scRNA-seq datasets from OS samples (*n* = 3), CS samples (*n* = 3) and CB tissue (*n* = 7). **C**, **D** Feature plots of mesenchymal cells from OS samples (*n* = 3), CS samples (*n* = 3) and CB tissue (*n* = 7). **E** Violin diagrams showed the expression of biomarkers of osteoblasts (COL1A1, BGLAP and RUNX2), chondrocytes (COL2A1, COMP and SOX9), sarcoma (POSTN and DCN) in cell subtypes of mesenchymal cells. **F** Bar plots show the proportion of various cell types in mesenchymal cells of OS, CS and CB samples. **G** Enriched signaling pathways were identified by GSVA enrichment analysis among these cell subtypes in mesenchymal cells. **H** Venn diagram showing the overlapped markers between DEGs from CS versus chondrocytes and DEGs from CS versus OS. **I** t-SNE plot and Bar plot showed the expression levels of KDELR1 in mesenchymal cells. **J, K** Volcano plots showed the DEGs in CS cells compared with chondrocytes or OS cells.

In order to illuminate the expression and transcriptional regulation characteristics of CS genes, we constructed the single-cell transcriptional landscape of CS by performing single-cell RNA sequencing (scRNA-seq) on CS and OS samples. And normal cancellous bone tissue was served as control bone (CB). A total of 102 380 cells were acquired after quality control exclusions and batch effect elimination. The Seurat package and Seurat alignment method canonical correlation analysis were used to normalize data, dimensionality reduction, clustering, differential expression and integrated analysis of datasets [29, 30]. Based on the expression of classic markers, mesenchymal cells (COL1A1, COL1A2), myeloid cells (CD14), osteoclasts (CTSK), T cells (CD3D), B and plasma cells (CD79A), vascular endothelial cells (PECAM1) and erythroid cells (HBB, HBA2) were identified (Fig. 1A, B; Figure S1A, B). We further reduced the dimensionality of mesenchymal cells to accurately identify and analyze CS cells, and its cell subtypes were identified including OS cells, CS cells, osteoblasts, adipocytes, chondrocytes, bone marrow derived mesenchymal stem cells (BMSCs) (Fig. 1C, D; Figure S1C). The most significant markers of the 6 clusters are shown in Figure S2A. Then, we analyzed the expression of biomarkers of osteoblasts (COL1A1, BGLAP and RUNX2), chondrocytes (COL2A1, COMP and SOX9) and sarcoma (POSTN and DCN), adipocyte (LPL and PPARG) and BMSCs (Fig. 1E; Figure S2B, C). Cell proportion analysis further clarified that CS cells mainly originate from CS samples, while OS cells originate from OS samples (Fig. 1F). GSVA analysis showed that CS was significantly enriched in pathways such as cartilage formation and differentiation, while OS was significantly enriched in osteogenic related pathways, further confirming the accuracy of the dimensionality reduction. Furthermore, we found that in tumor-related pathways such as DNA replication, the enrichment intensity of CS was weaker than that of OS, indicating a lower malignancy level of CS. Besides, ECM secretion and Golgi-ER-related protein synthesis were enriched in CS (Fig. 1G). Subsequently, we analyzed CS-specific differentially expressed genes (DEGs), a total of 455 and 145 DEGs were identified in CS versus chondrocytes and CS versus OS, respectively. Furthermore, we identified the overlapped markers between two list of DEGs, in which KDELR1 came out in front (Fig. 1H). It was consistent with the expression levels of KDELR1 in mesenchymal cells (Fig. 1I). Volcano plots showed that KDELR1 was elevated in CS cells compared with both chondrocytes and OS cells (Fig. 1J, K). These results demonstrated that KDELR1 is a CS-specific molecule and might be involved in the pathogenesis of CS.

### Elevated expression of KDELR1 is linked to high-grade CS

**Fig. 2:**
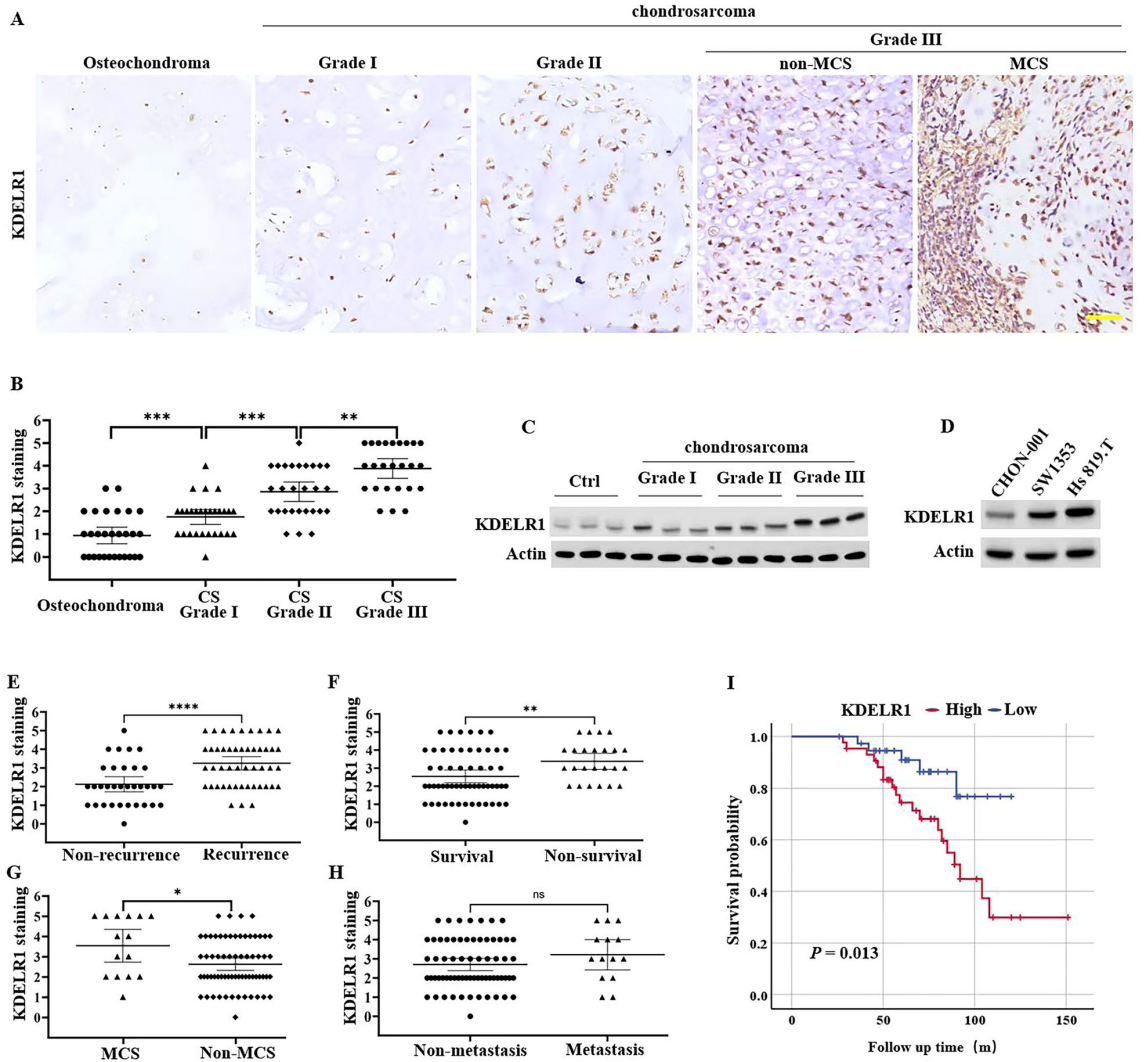
KDELR1 is malignancy-related in CS. **A** Representative images of IHC staining for KDELR1 in osteochondroma and CS specimens with various grades. **B** The quantification and analysis of KDELR1 between these specimens with various grades in IHC staining according to the percentage of positive cells. **P < 0.01, ***P < 0.001. **C** Western blot of KDELR1 in osteochondroma and CS specimens with various grades. **D** Western blot of KDELR1 in human normal cartilage cell line CHON-001, and CS cell lines SW1353 and Hs 819.T. The quantification and analysis of KDELR1 in IHC staining between groups stratified by recurrence (**E**), survival (**F**), MCS (**G**) and metastasis (**H**). ns *P* > 0.05, *P < 0.05, **P < 0.01, ****P < 0.0001. **I** Kaplan-Meier survival probability curves of CS patients with high or low expression of KDELR1. *P* = 0.013.

Initially, the expression of KDELR1 in CS was examined in 110 samples, comprising osteochondroma specimens (*n* = 29), and CS specimens with various grades including Grade I (*n* = 28), Grade II (*n* = 28), Grade III (*n* = 25). The results of IHC staining of these specimens exhibited that the expression level of KDELR1 positively correlated with the grade malignancy of CS (Fig. 2A, B). The Western blot results aligned with the IHC findings (Fig. 2C). We also determined the expression of KDELR1 in human normal cartilage cell line CHON-001, and CS cell lines SW1353 and Hs 819.T. The results showed a higher expression of KDELR1 in SW1353 and Hs 819.T CS cells than CHON-001 cells (Fig. 2D). We further detected that KDELR1 was also correlated with other indicators that can reflect the malignancy of CS, including recurrence (Fig. 2E), survival (Fig. 2F), MCS (Fig. 2G), and metastasis (Fig. 2H). Finally, we analyzed the relationship of KDELR1 to Kaplan-Meier free survival of patients. Patients with higher expression levels of KDELR1 had a poorer prognosis; this fact strongly supports the role of KDELR1 in the disease progression (Fig. 2I). All these results indicated that the expression level of KDELR1 can reflect the malignancy extent of CS.

### KDELR1 influences malignant behaviors of CS

**Fig. 3:**
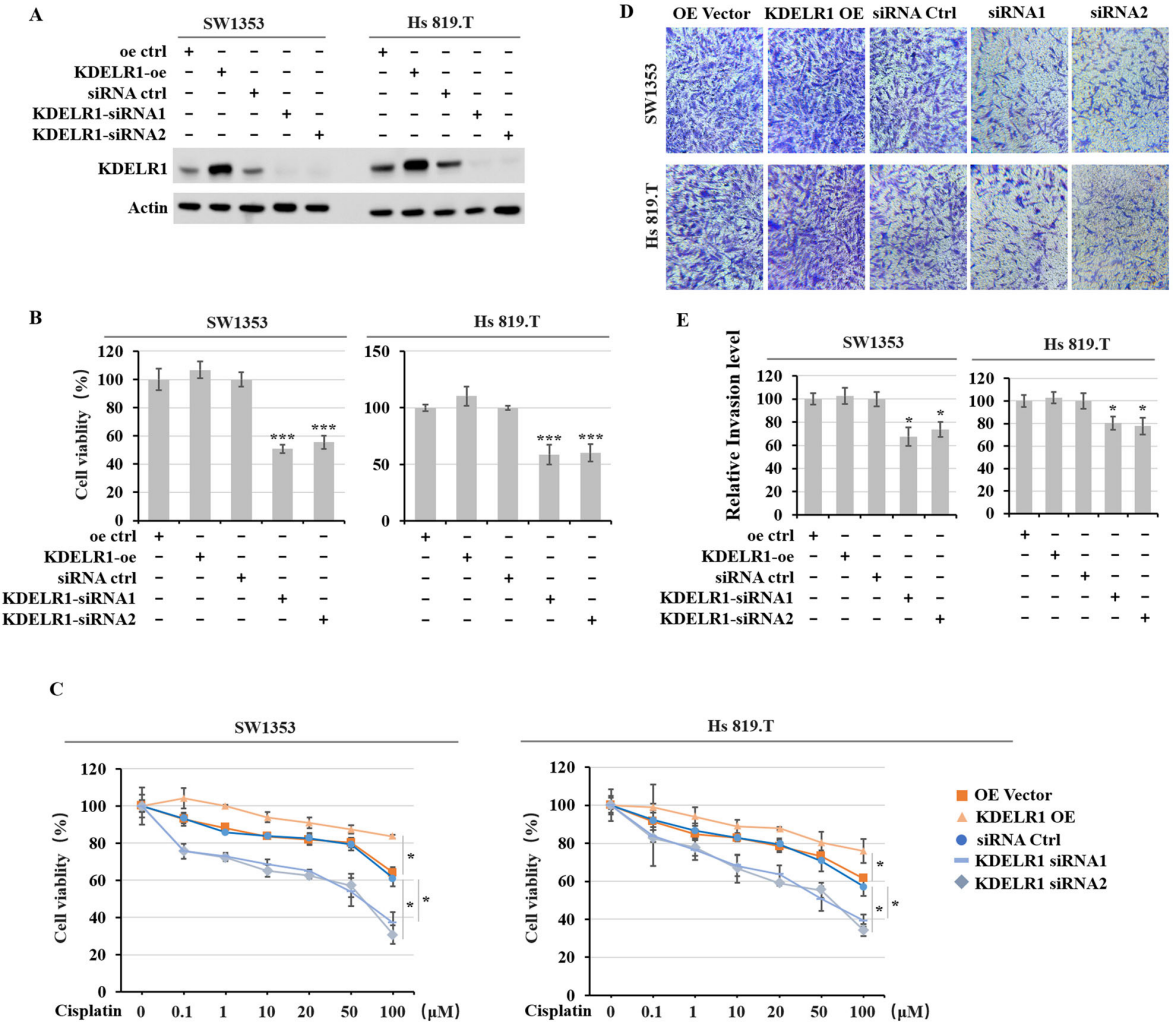
Elevated KDELR1 is closely related to malignant behaviors of CS. **A** Western blot of KDELR1 expression in SW1353 and Hs 819.T sublines infected by lentivirus of oe-ctrl, KDELR1-oe, siRNA-ctrl, KDELR1-siRNAl, or KDELR1-siRNA2. **B** CCK-8 assay detected the cell viability of SW1353 and Hs 819.T sublines in **A**. ***P < 0.001. **C** CCK-8 assay detected the cell viability of these SW1353 and Hs 819.T sublines stimulated with a dose gradient of cisplatin from 0 to 100 uM. **D** Migration assay of these sublines in **A**. **E** Migration assay detected the relative invasion level of these sublines in **A**. *P < 0.05.

To further investigate the impact of KDELR1 on CS, we generated CS sublines that overexpressed KDELR1, or siRNA targeted KDELR1, or control. The transfection efficiency was verified by western blot (Fig. 3A). Then, CCK8 assay revealed that cell viability of CS cells was not affected by overexpressed KDELR1 but was dramatically suppressed in KDELR1-kd CS cells (Fig. 3B). Cisplatin plays a key component of CS chemotherapy drugs in the systemic therapy of CS [31, 32]. Considering the close relationship between membrane proteins and tumor drug resistance, and KDELR1’s involvement in the process of membrane proteins modification and processing, we further explored how KDELR1 regulates the chemotherapeutic effect of Cisplatin in CS therapy. We found that KDELR1-kd had actions of promoting sensitivity of CS cells to Cisplatin and reduced Cisplatin-resistance in CS therapy (Fig. 3C). Then, transwell assay showed that KDELR1-oe has no effect on CS migration, but KDELR1-kd attenuates the migratory ability of CS (Fig. 3D, E). Together, these data collectively prove that KDELR1 is closely related to malignant behaviors and chemotherapy resistance of CS.

### Knocking down KDELR1 leads the YAP1 accumulation in cytoplasm and its inactivation

**Fig. 4:**
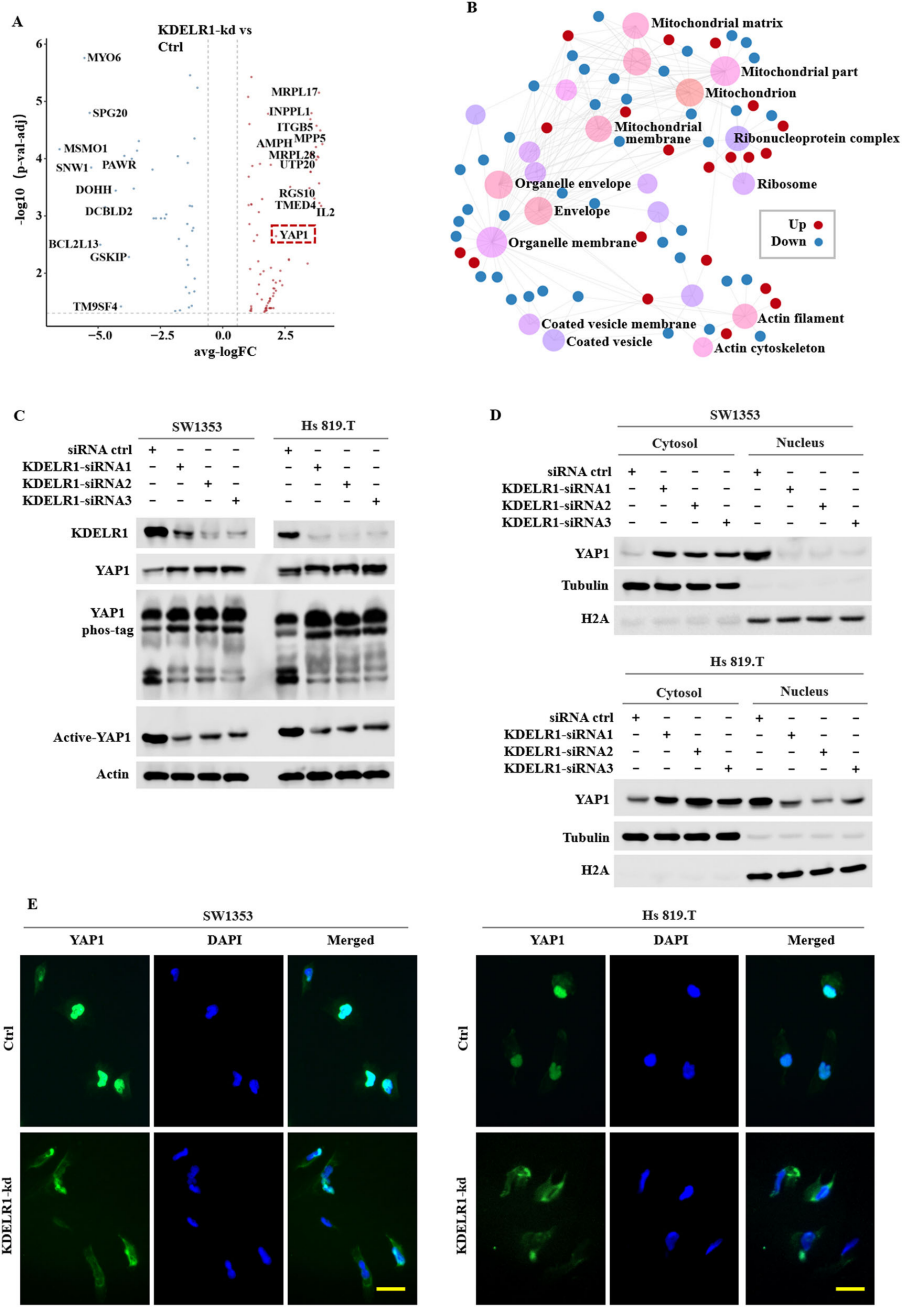
KDELR1-kd promotes YAP1 phosphorylation and inhibits its nuclear translocation. **A** Volcano plot of differently expression proteins between KDELR1-kd and ctrl-siRNA SW1353 CS cells determined by LC-MS analysis. **B** GO enrichment analysis of these DEGs in **A**. **C** Western blot of KDELR1, YAP1, YAP1 phos-tag, active-YAP1 in SW1353 and Hs 819.T sublines infected with KDELR1 siRNA (KDELR1-siRNA1, KDELR1-siRNA2, or KDELR1-siRNA3) or ctrl-siRNA. **D** Western blot of YAP1 expression in cytoplasm and nucleus of these SW1353 and Hs 819.T sublines in **C**. **E** IF assay of YAP1 expression and location in KDELR1-kd SW1353 and Hs 819.T CS cells.

KDELR1 recognizes, binds, and subsequently retrieves the HDEL-containing protein from Golgi to endoplasmic reticulum, thus regulating the synthesis and processing of membrane proteins [33]. Therefore, we utilized LC-MS analysis to explore the downstream regulators of KDELR1. The volcano plot in Fig. 4A shows that YAP1 was one of the proteins with the most significant expression differences between KDELR1-kd and ctrl-siRNA SW1353 cells. GO enrichment analysis indicated that KDELR1 was associated with the expression of membrane protein and secretory protein involving in components such as ribonucleoprotein complex, coated vesicle membrane, and actin cytoskeleton (Fig. 4B). Dephosphorylated YAP1 translocates from cytoplasm to nucleus and targets TEA domain (TEAD) transcription factor family to regulate the expression of its targeted genes, while phosphorylation deactivates YAP1 and promotes its cytoplasmic localization. Therefore, we first detected the expression of different forms of YAP1 in KDELR1-kd and ctrl-siRNA SW1353 and Hs 819.T cells. The results showed knocking down KDELR1 upregulated the total YAP1 and phosphorylated YAP1 and suppressed the active-YAP1 (Fig. 4C). For further confirmation, the nuclear and cytoplasmic protein were extracted and its total YAP1 was determined. The results demonstrated that KDELR1-kd decreased dephosphorylated YAP1 in nucleus, meanwhile upregulated phosphorylated YAP1 in cytoplasm (Fig. 4D). Then, IF assay was conducted and the figures illustrate that KDELR1-kd elevated p-YAP1 and led its elevation of cytoplasmic accumulation (Fig. 4E). All this showed that KDELR1-kd promoted YAP1 phosphorylation, which inhibited its nuclear translocation and subsequently caused its accumulation and deactivation.

### Knocking down KDELR1 enhances Hippo-Yap signaling pathway

**Fig. 5:**
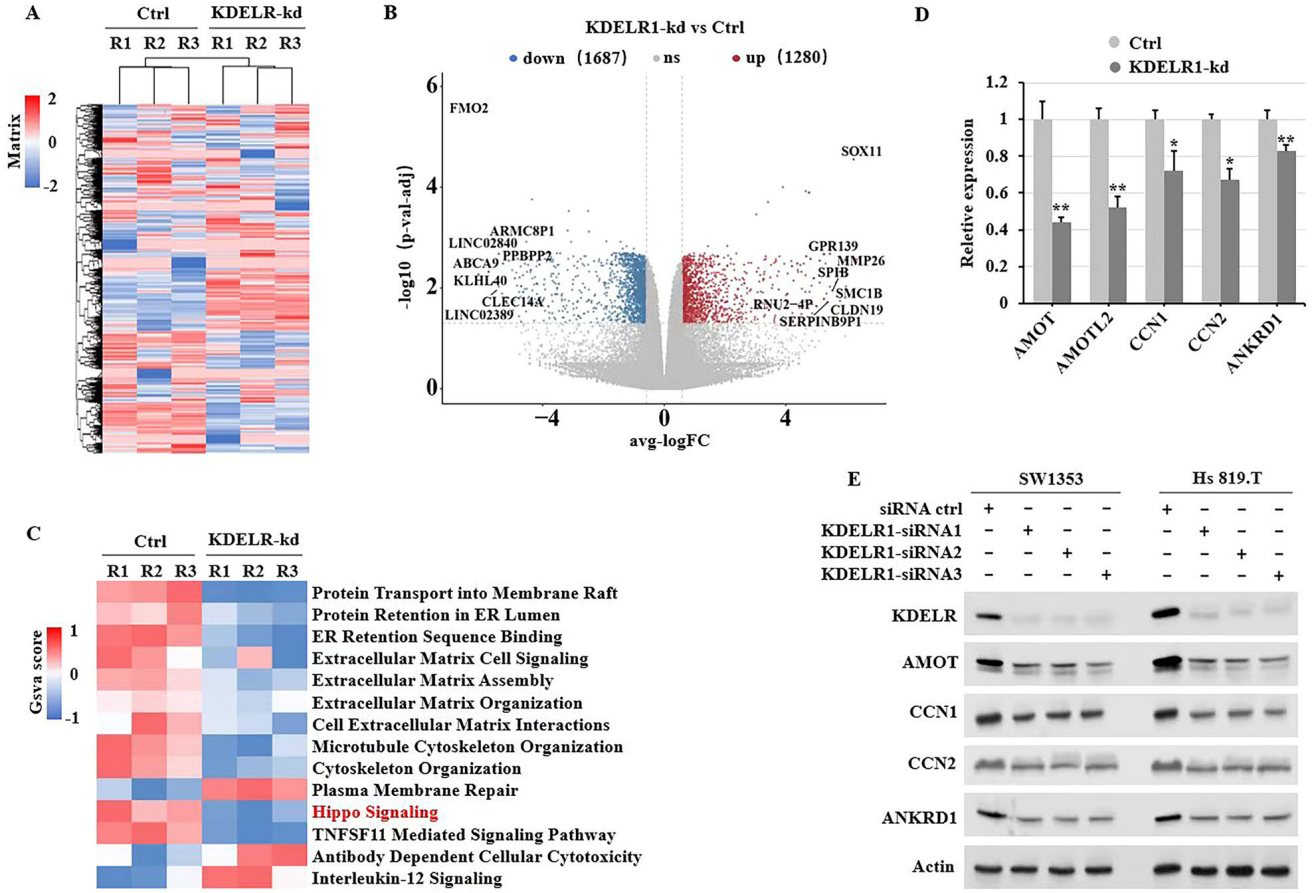
KDELR1-kd mediates Hippo-YAP signaling pathway. Heap map (**A**) and volcano plot (**B**) of different expression genes between KDELR1-kd and ctrl-siRNA SW1353 CS cells determined by RNA-seq. **C** Function enrichment of these different expression genes in **A**. (**D**) qRT-PCR of AMOT, AMOTL2, CCN1, CCN2 and ANKRD1 mRNA expression in KDELR1-kd and ctrl-siRNA SW1353 CS cells. *P < 0.05, **P < 0.01. **E** Western blot of AMOT, AMOTL2, CCN1, CCN2 and ANKRD1 expression in SW1353 and Hs 819.T sublines infected with KDELR1 siRNAs or ctrl-siRNA.

To elucidate the YAP1-phosphorylation mechanism of knocking down KDELR1, the total RNA of KDELRI-kd and siRNA-ctrl cells were extracted and DEGs were analyzed by RNA-seq. The DEGs are illustrated in Fig. 5A, B. Functional enrichment analysis was conducted, revealing that, in addition to the processes and pathways related to the processing of membrane and secreted proteins, the Hippo-YAP1 signaling pathway may play a significant role in the accumulation and inactivation of YAP1 (Fig. 5C). Next, the data of qRT-PCR and western blot exhibited a lower mRNA and protein expression of these downstream targets of Hippo-YAP1 signaling pathway comparing to siRNA-ctrl cells (Fig. 5D, E). These findings suggest that KDELR1 plays a significant role in modulating the Hippo-YAP signaling pathway.

### The regulation of Hippo-YAP pathway by KDELR1 is achieved by promoting the expression of MAP4K4

**Fig. 6:**
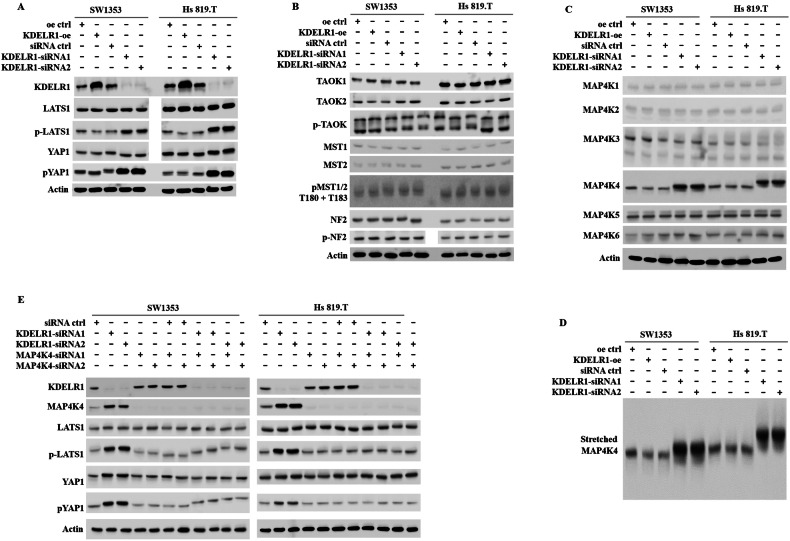
KDELR1-kd enhances Hippo-YAP1 signal pathway by YAP1 phosphorylation via MAP4K4. **A**–**C** Western blot of KDELR1, LATS1, p-LATS1, YAP1, p-YAP1, TAOK1, TAOK2, p-TAOK, MST1, MST2, p-MST1/2, NF2, p-NF2, MAP4K1/2/3/4/5/6 in SW1353 and Hs 819.T sublines of oe-ctrl, KDELR1-oe, siRNA-ctrl, KDELR1-siRNAl, or KDELR1-siRNA2. **D** The “Stretched MAP4K4” image was the vertical extension version of the immunoblotting image of MAP4K4 in C to visualize the expression and mobility shift of MAP4K4 clearly. **E** Western blot of KDELR1, MAP4K4, LATS1, p-LATS1, YAP1, p-YAP1 in SW1353 and Hs 819.T sublines of siRNA-ctrl, KDELR1-siRNAl, KDELR1-siRNA2, MAP4K4-siRNAl, or MAP4K4-siRNA2.

Subsequently, we delved deeper into understanding how KDELR1 influences the Hippo-YAP signaling pathway. As a downstream nuclear effector of the mammalian Hippo signaling pathway, YAP/TAZ was regulated by TAO kinases (TAOKs), large tumor suppressor 1/2 (LATS1/2), mammalian STE20-like protein kinase (MST1/2), p-MST1/2, MAP4Ks and NF2-depended MAP4K 4/6 [34]. Thus, we performed a comprehensive detection of YAP-related kinases in KDELR1-oe and KDELR1-kd CS cells, which showed that the YAP1-phosphorylation was not regulated by TAOKs, MST1/2, and the scaffold proteins NF2, but by p-LATS1 and MAP4K4 among the MAP4Ks family (Fig. 6A-D). Furthermore, we found that reduced KDELR1 promoted the expression of MAP4K4 and the phosphorylation of LATS1 and YAP1 in CS cells (Fig. 6E). The above results revealed that MAP4K4 may serve as a pivotal kinase in mediating KDELR1’s impact on Hippo-YAP signaling.

### KDELR1 regulates the expression of MAP4K4 by affecting integrins

**Fig. 7:**
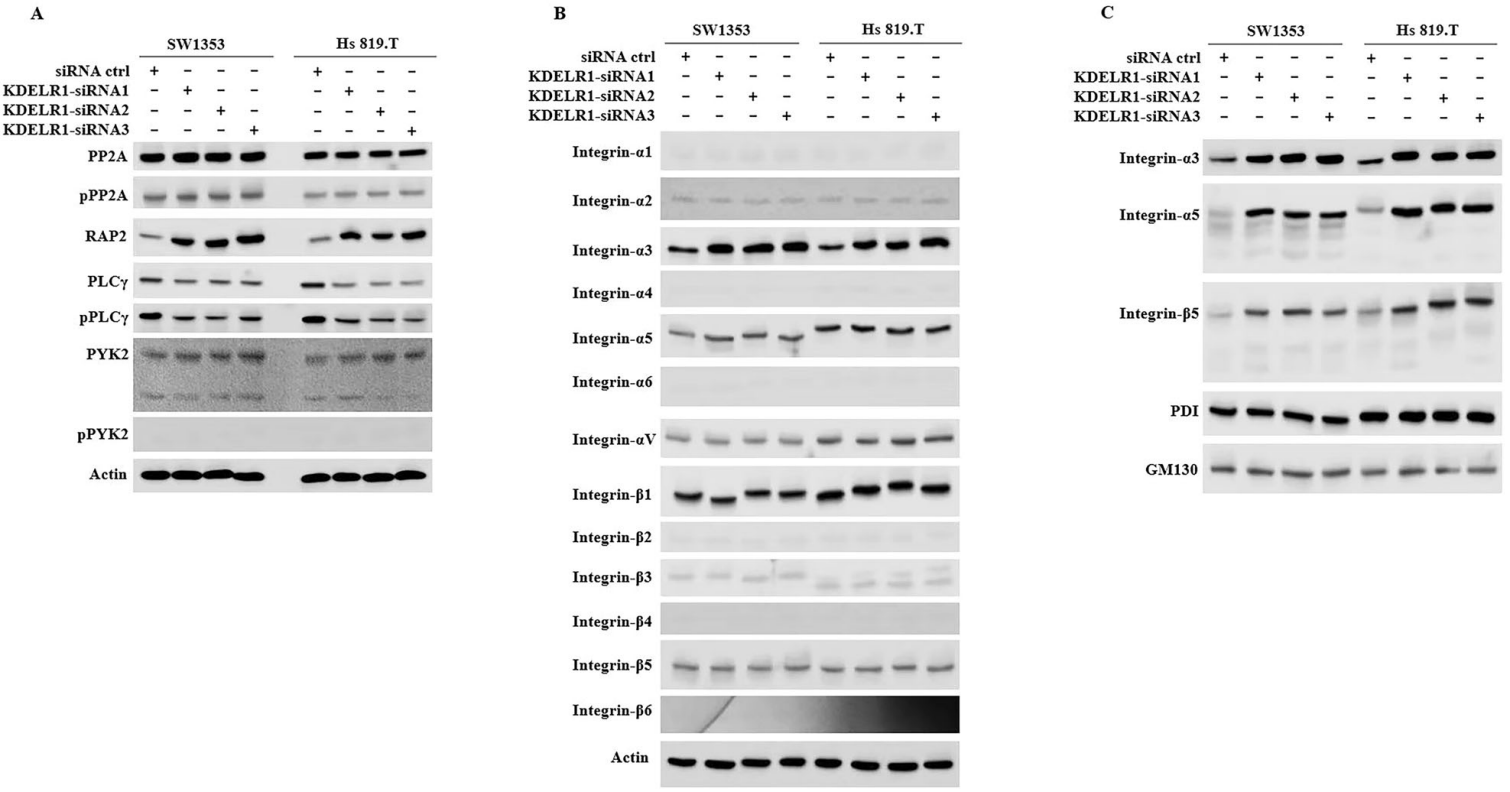
MAP4K4-regulation of KDELR1 was mediated by integrins. **A** and **B** Western blot of PP2A, p-PP2A, RAP2, PLCγ, p-PLCγ, PYK2, p-PYK2 and integrins expression in SW1353 and Hs 819.T sublines infected with KDELR1 siRNAs or ctrl-siRNA. **C** Western blot of integrin-α3, integrin-α5 and integrin-β5 expression in ER and Golgi of these SW1353 and Hs 819.T sublines in A.

MAP4K4 belongs to the serine/threonine protein kinase family, not a membrane or secreted protein. Therefore, we analyzed KDELR1 mediated for regulating MAP4K4 in CS. The upstream regulators of MAP4K4 including protein phosphatase 2 A (PP2A), RAP2, phospholipase Cγ (PLCγ), protein-tyrosine kinase 2 (PYK2) have been well-proved [35, 36]. We found that PLCγ and RAP2 were the key regulators of KDELR1-kd-induced MAP4K4 (Fig. 7A). The general function of RAP2 was regulating cell adhesion and spreading mediated by integrin [37]. Besides, PLCγ is a critical part of integrin-mediated transduction pathways [38]. Notably, as membrane proteins, Integrins are synthesized and processed by the ER-Golgi system and could be subject to regulation by KDELR1. Integrins, in turn, are crucial signaling molecules implicated in tumor aggressiveness and resistance to drugs. We performed a comprehensive detection of integrin family in KDELR1-kd and siRNA-ctrl CS cells; the results showed that integrin-α3 was upregulated by KDELR1-kd (Fig. 7B). Furthermore, we isolated the ER and Golgi and subsequently the expression of integrins family in ER and Golgi were measured. The results exhibited that integrin-α3, integrin-α5 and integrin-β5 were trapped in ER and Golgi (Fig. 7C). In summary, these results showed that the MAP4K4-regulation of KDELR1 was mediated by affecting the synthesis, processing, and plasma membrane translocation of integrin-α3, integrin-α5 and integrin-β5.

### KDELR1 knockdown impedes human CS cells progression in vivo

**Fig. 8:**
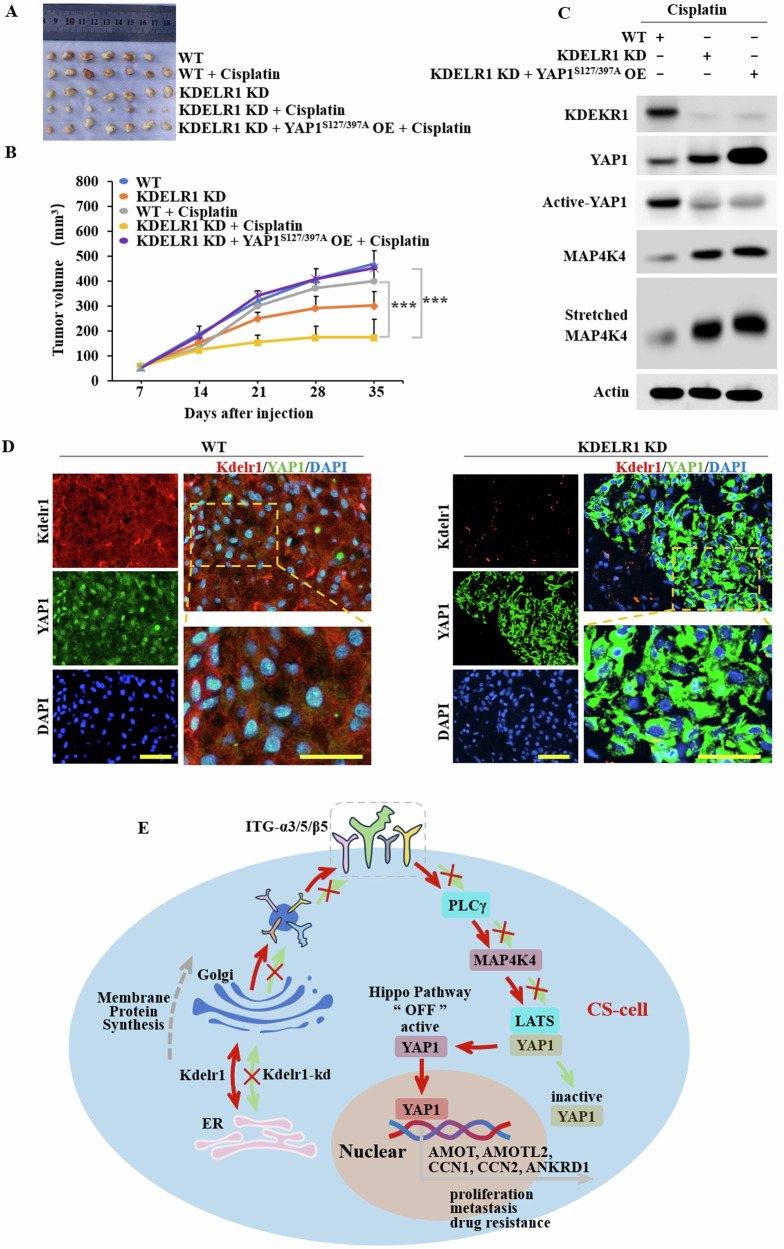
KDELR1-kd inhibits human CS cells progression. **A** Photographs of tumors excised from groups of WT mice (*n* = 6), WT+cisplatin (*n* = 7), KDELR1-kd (*n* = 7), KDELR1-kd+cisplatin (*n* = 7) and KDELR1-kd + (YAP1^S127/397A^ OE) +cisplatin (*n* = 7). **B** Size growth 35 days from initial tumor detection. ***P < 0.001. **C** Western blot of KDELR1, YAP1, active-YAP1, MAP4K4 and stretched MAP4K4 in these xenograft tumors of WT and KDELR1-kd. **D** IF staining of KDELR1 and YAP1 in these tumors. **E** Schematic diagram showing the role of KDELR1 in CS proliferation, metastasis, and drug-resistance.

To further substantiates the role of KDELR1 in regulating drug resistance and malignant behavior in CS. we established subcutaneous tumor xenograft models. The tumors were excised, collected, photographed (Fig. 8A) and sectioned after euthanasia. KDELR1-kd reduced Cisplatin-resistance in CS therapy, which was inhibited by overexpressed active-YAP1 (YAP1^S127/397A^ OE) (Fig. 8B). The protein of parts of tumors was extracted, and the expression of MAP4K4, YAP1 and active-YAP1 was determined. Active-YAP1 was overexpressed stably at 35 days in YAP1^S127/397A^ OE group. The results confirmed that KDELR1-kd-led YAP1-phosphorylation were associated with increased MAP4K4 (Fig. 8C). The results of IF staining showed that the expression of YAP1 was significantly increased in KDELR1-kd tumor xenograft (Fig. 8D). Taken together, the results of these in vivo studies confirmed that KDELR1-kd ameliorates CS proliferation, metastasis, and drug-resistance by enhancing Hippo-YAP1signaling pathway via integrin-α3/5β5-PLCγ-MAP4K4 axis.

## Discussion

Limited research exists on single-cell sequencing of CS, with only one study exploring the role of endoplasmic reticulum stress in tumor promotion [39]. Our study utilized single-cell sequencing to analyze 3 CS samples, 3 OS samples, and 7 normal cancellous bone samples, creating a single-cell transcription map. Through re-clustering of the mesenchymal portion, we identified six cell subpopulations based on markers. Subsequent GSVA analysis revealed significant activation of bone mineralization pathways in OS cells, while cartilage and osteogenesis-related pathways were primarily activated in CS cells. OS exhibited stronger DNA replication function compared to CS. Notably, CS cells displayed high activation of ECM secretion, Golgi apparatus-related proteins, and endoplasmic reticulum function. Additionally, the expression of KDELR1 was notably higher in CS compared to OS and chondrocytes, as determined by intersecting gene analysis.

Recently, many researchers have focused on Hippo-YAP signaling pathway in search of novel perspectives and therapeutic potential of cancers. Enhancement of Hippo-YAP signaling pathway and its key components, including LATS1/2 and MST1/2, were involved in tumorigenesis, proliferation, invasion, migration, and drug resistance of multiple types of malignancies, such as lung, small intestine, breast and colon cancers [40]. Joel D. Pearson and his colleagues classified pan-cancer into two categories: YAP deficient and YAP positive cancers. Distinct YAP-dependent pharmaceutical vulnerabilities were found between cancers in different categories [41], indicating that YAP is the key to these cancers’ resistance and malignancy. This is consistent with our study results showing Hippo-YAP signaling pathway function on drug-resistance of CS and, to our knowledge, this is the first study revealing the vital role of Hippo-YAP signaling pathway in drug-resistance of CS cells and its specific molecular mechanisms.

Hippo-YAP signaling pathway has also been related to the proliferation, invasion, and tumorigenesis of CS [14, 15, 42], which was in accordance with our study. Besides pathogenesis and aggressiveness, decreased YAP/TAZ and LATS1 promoted the cell cycle arrest, senescence, and apoptosis of CS [43, 44]. Although the crucial role of Hippo-YAP signaling pathway in CS was well-documented, the underlying causes and mechanisms of excessive suppression of Hippo-YAP signaling pathway (Hippo pathway “OFF”) in CS cells needs further investigation. More importantly, several types of drugs target the Hippo/YAP signaling pathway, including tyrosine kinase inhibitors (Dasatinib), Doxorubicin and Cisplatin. These agents have either already been launched or are in the process of being introduced for the treatment of CS. Therefore, identifying therapeutic targets of Hippo/YAP signaling pathway could help both inhibit the progression of CS, and enhance the sensitivity of these drugs to CS.

Upstream pathways that regulate the ON/OFF switch of Hippo-YAP signaling pathway constitute an intricate signals network, in which RhoA, a small GTPases of Ras superfamily Rho GTPases, participate in regulating YAP dephosphorylation induced by cell attachment [40, 45]. ECM stiffness alteration is an important mechanical signal. Ras related GTPase RAP2 can be activated by low ECM stiffness, leading to the activation of LATS1/2 [46]. In addition, Agrin, as an ECM protein polysaccharide, transmits signals to the Hippo pathway by inhibiting the function of LATS1/2 [47]. As an evolutionarily conservative pathway, Hippo/YAP signaling pathway was induced by these signals to regulate cell proliferation, apoptosis, stem cell self-renewal, and multiple biological functions such as tissue development and regeneration [40]. These stimulations and its complex regulation for comprehensive function of Hippo/YAP signaling pathway reiterate the complexity of the regulating signals network.

In conclusion, we demonstrated that KDELR1, one of KDEL receptors, plays a pivotal role in proliferation, migration, metastasis, and drug-resistance of CS. Furthermore, we showed that KDELR1 knockdown and consequent inhibition of the malignant behaviors of CS, is achieved by promoting the expression of MAP4K4 via ITG-α3/5β5-PLCγ followed by YAP1 accumulation in cytoplasm and its inactivation, and subsequent Hippo-YAP1 signaling pathway. Manipulating KDELR1 expression inhibited malignant behaviors and drug-resistance of CS through the enhancement of Hippo-YAP1signaling pathway via ITG-α3/5β5-PLCγ-MAP4K4 axis. KDELR1 is a dependable biomarker of CS malignancy degree and prognosis and a novel and promising therapeutic target for CS.

## Supplementary information

Antibody Information

Figure S1 and S2

Original western blots

## Data Availability

The authors declare that all relevant data of this study are available within the article and its supplementary files or from the corresponding author on reasonable request.
