# Supplementary material for: KDELR1 regulates chondrosarcoma drug resistance and malignant behavior through Intergrin-Hippo-YAP1 axis
Source: Cell Death Dis. 2024 Dec 23;15(12):928. doi: 10.1038/s41419-024-07264-7 (PMC11666724; doi:10.1038/s41419-024-07264-7)
Supplement: Supplementary file 1 — Antibody Information [file 41419_2024_7264_MOESM1_ESM.docx]

Table S1. Antibody Information

| antibody | company | item |
| --- | --- | --- |
| TAOK1 | Abcam | ab197891 |
| TAOK2 | Abcam | ab254136 |
| pTAOK | Abcam | ab248030 |
| MST2 | Abcam | ab79199 |
| LATS1 | Abcam | ab243656 |
| pLATS1 | Abcam | ab305029 |
| YAP1 | Abcam | ab52771 |
| NF2 | Abcam | ab308025 |
| pNF2 | Abcam | ab2478 |
| MAP4K1 | Abcam | ab33910 |
| MAP4K2 | Abcam | ab184169 |
| MAP4K3 | Cell Signaling Technology | 92427S |
| MAP4K4 | Abcam | ab155583 |
| MAP4K5 | Abcam | ab96551 |
| MAP4K6 | Abcam | ab154256 |
| PP2A | Abcam | ab32065 |
| pPP2A | Abcam | ab314196 |
| RAP2 | Abcam | ab173296 |
| PYK2 | Abcam | ab226798 |
| pPYK2 | Abcam | ab131543 |
| PLCγ1 | Abcam | ab302940 |
| pPLCγ1 | Abcam | ab76031 |
| Integrin α1 | Abcam | ab181434 |
| Integrin α2 | Abcam | ab271936 |
| Integrin α3 | Abcam | ab131055 |
| Integrin α4 | Abcam | ab75760 |
| Integrin α5 | Abcam | ab150361 |
| Integrin α6 | Abcam | ab308050 |
| Integrin αV | Abcam | ab179475 |
| Integrin β1 | Abcam | ab52971 |
| Integrin β2 | Abcam | ab307406 |
| Integrin β3 | Abcam | ab179473 |
| Integrin β4 | Abcam | ab133682 |
| Integrin β5 | Abcam | ab309092 |
| Integrin β6 | Abcam | ab187155 |
| PDIA2 | Abcam | ab223520 |
