# Supplementary material for: KDELR1 regulates chondrosarcoma drug resistance and malignant behavior through Intergrin-Hippo-YAP1 axis
Source: Cell Death Dis. 2024 Dec 23;15(12):928. doi: 10.1038/s41419-024-07264-7 (PMC11666724; doi:10.1038/s41419-024-07264-7)
Supplement: Supplementary file 2 — Figure S1 and S2 [file 41419_2024_7264_MOESM2_ESM.pdf]

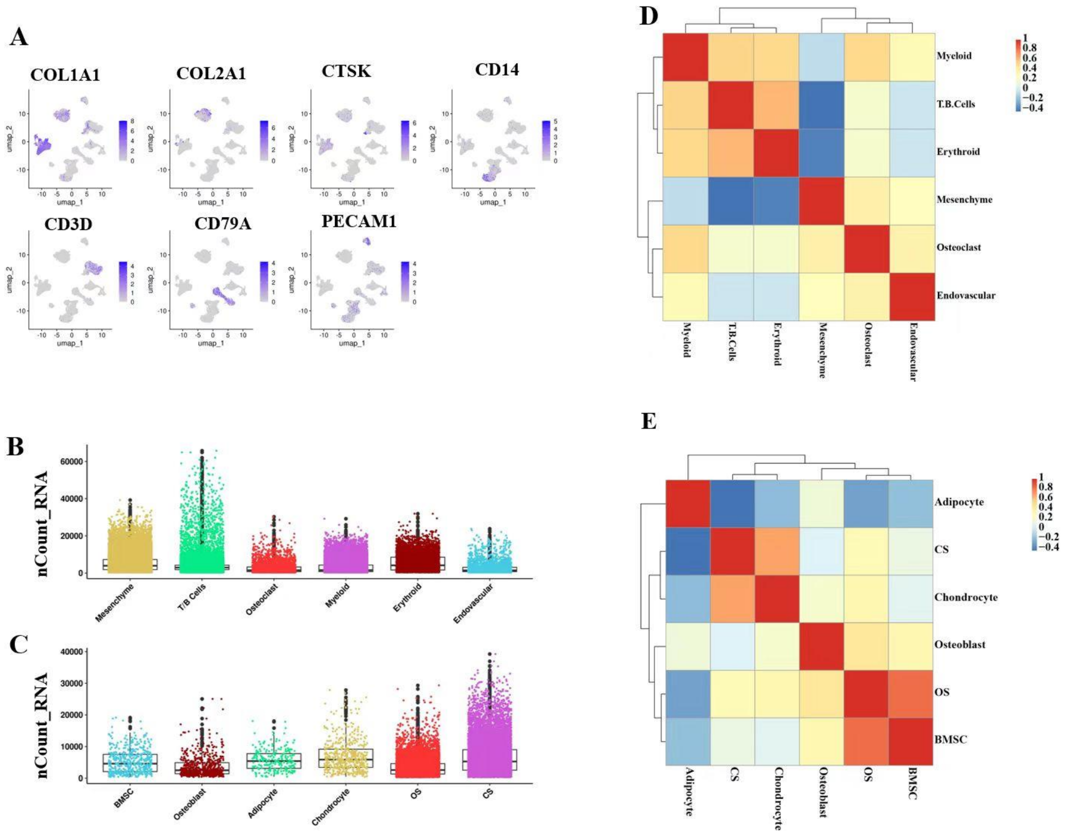

A
COL1A1
COL2A1
CTSK
CD14
CD3D
CD79A
PECAM1
umap_1
umap_2
B
nCount_RNA
Mesenchyme
T/B Cells
Osteoclast
Myeloid
Erythroid
Endovascular
C
nCount_RNA
BMSC
Osteoblast
Adipocyte
Chondrocyte
OS
CS
D
Myeloid
T.B.Cells
Erythroid
Mesenchyme
Osteoclast
Endovascular
E
Adipocyte
CS
Chondrocyte
Osteoblast
OS
BMSC

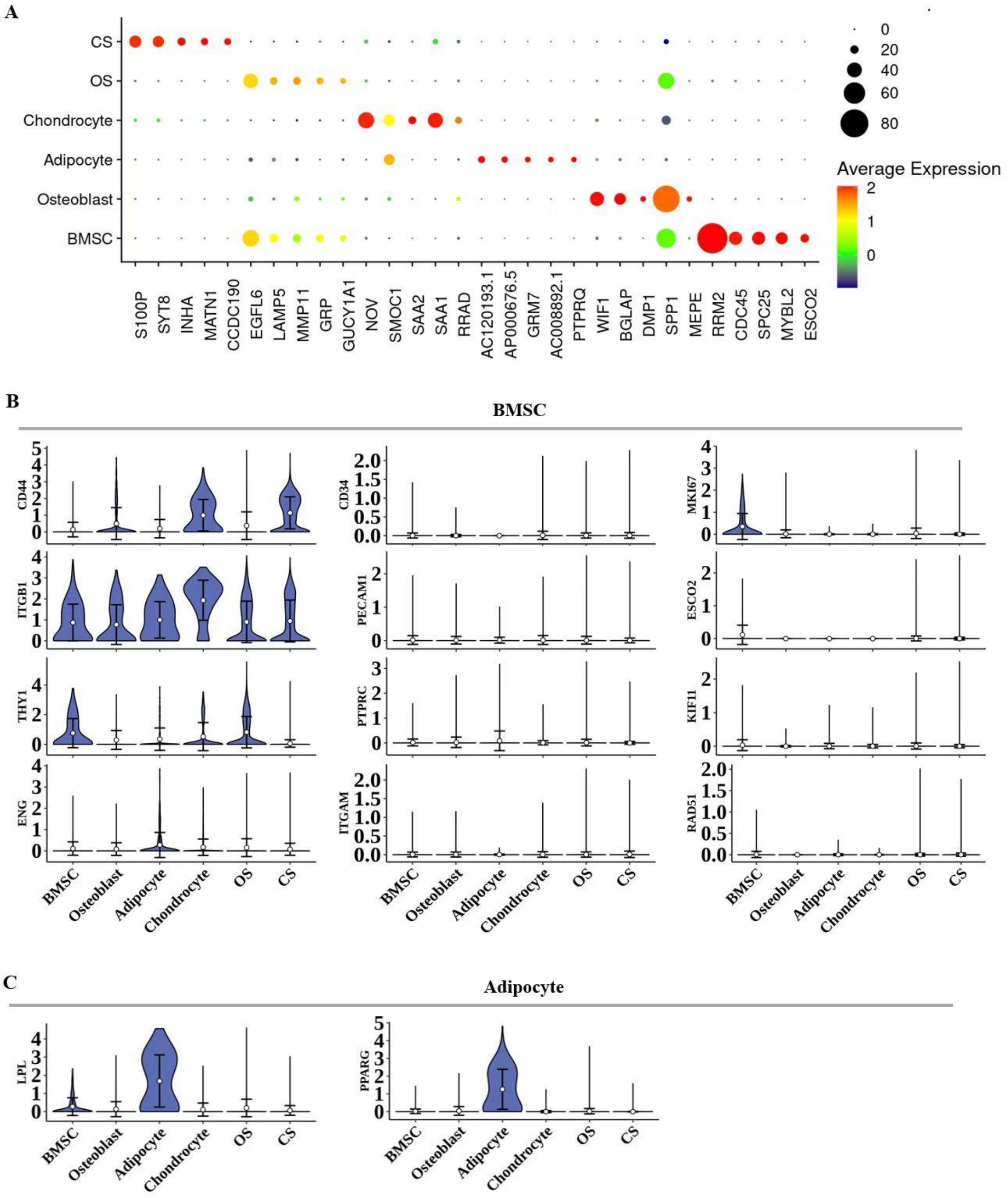

Figure S1. (A) The expression patterns of genes markers in the UMAP visualization. (B-C) The total number of molecules detected in cells. (D-E) Heatmap of intercellular communication among cell subtypes.

Figure S2. (A) Dot-plot heatmap of the genes markers in mesenchymal cells. (B) Violin plots showing differential expression of BMSC markers in each mesenchymal cluster. (C) Violin plots showing differential expression of Adipocyte markers in each mesenchymal cluster.
