# Supplementary material for: KDELR1 regulates chondrosarcoma drug resistance and malignant behavior through Intergrin-Hippo-YAP1 axis
Source: Cell Death Dis. 2024 Dec 23;15(12):928. doi: 10.1038/s41419-024-07264-7 (PMC11666724; doi:10.1038/s41419-024-07264-7)
Supplement: Supplementary file 3 — Original western blots [file 41419_2024_7264_MOESM3_ESM.pptx]

## Slide 1
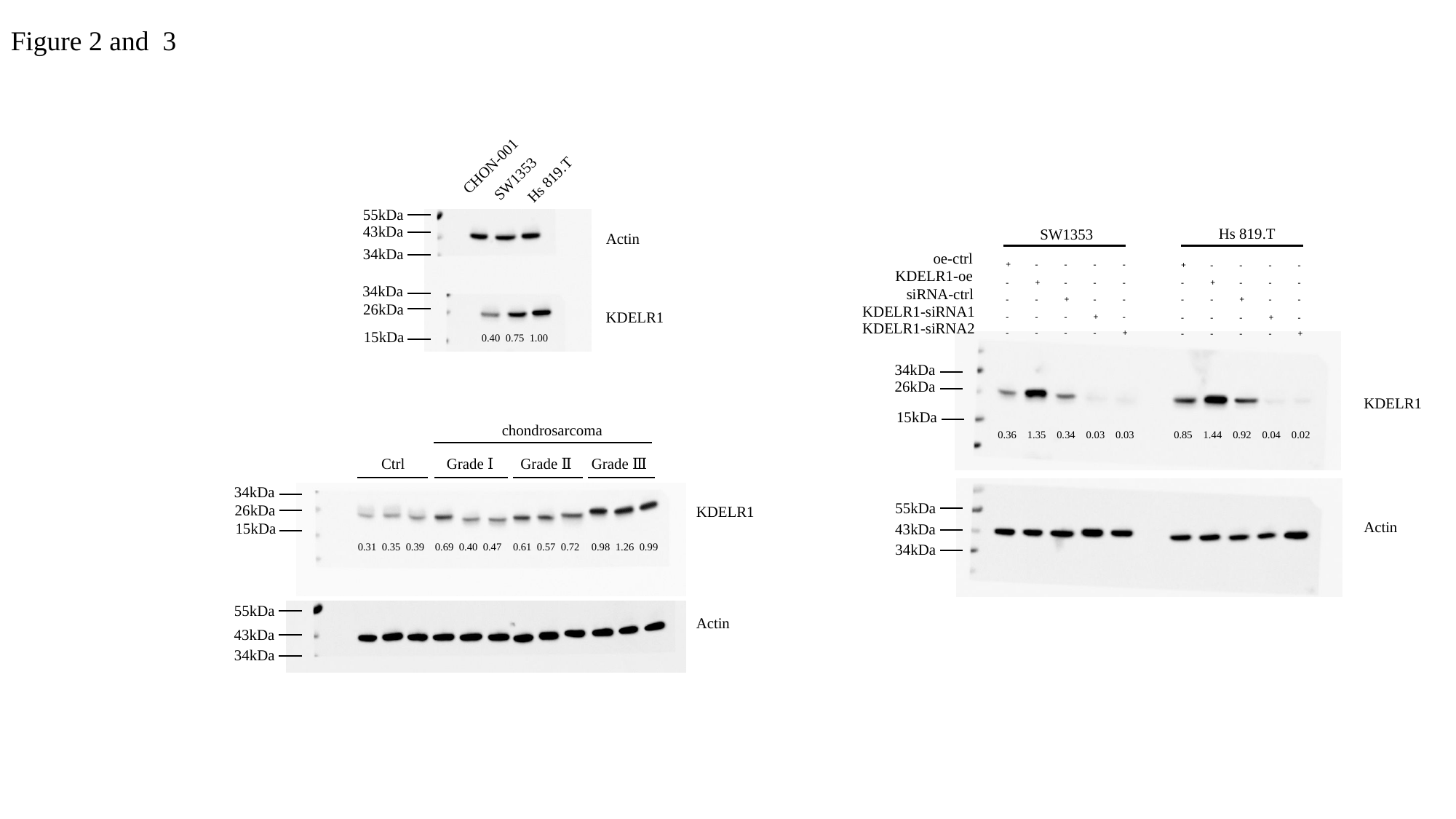

Figure 2 and 3
CHON-001
Hs 819.T
SW1353
55kDa
43kDa
Hs 819.T
SW1353
Actin
34kDa
+
-
-
-
-
oe-ctrl
+
-
-
-
-
KDELR1-oe
-
+
-
-
-
-
+
-
-
-
34kDa
-
-
+
-
-
-
-
+
-
-
siRNA-ctrl
26kDa
-
-
-
+
-
KDELR1-siRNA1
-
-
-
+
-
KDELR1
-
-
-
-
+
-
-
-
-
+
KDELR1-siRNA2
15kDa
0.40 0.75 1.00
34kDa
26kDa
KDELR1
15kDa
chondrosarcoma
0.36 1.35 0.34 0.03 0.03
0.85 1.44 0.92 0.04 0.02
Ctrl
Grade Ⅰ
Grade Ⅱ
Grade Ⅲ
34kDa
55kDa
26kDa
KDELR1
Actin
15kDa
43kDa
34kDa
0.31 0.35 0.39
0.69 0.40 0.47
0.61 0.57 0.72
0.98 1.26 0.99
55kDa
Actin
43kDa
34kDa

## Slide 2
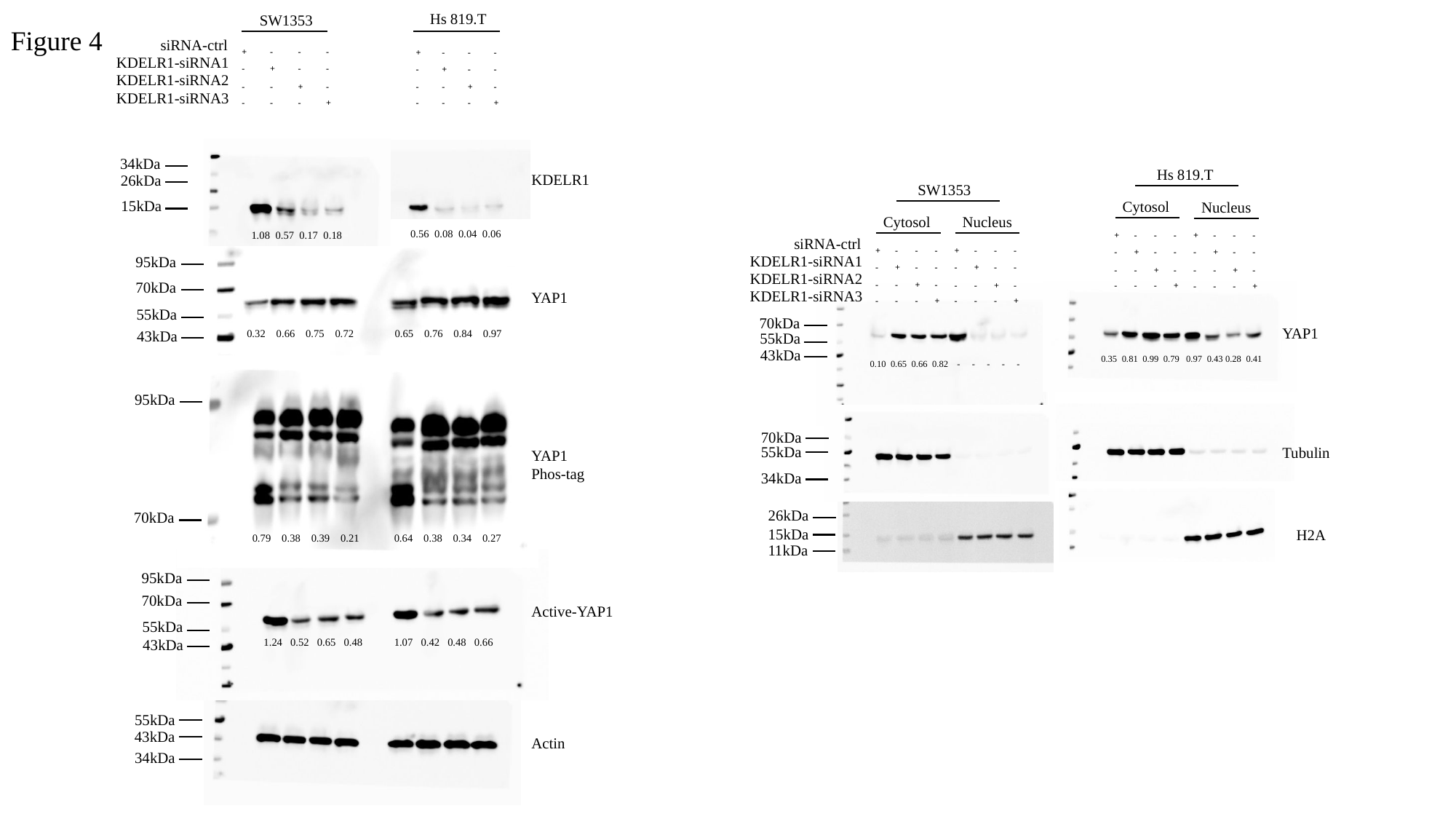

Hs 819.T
SW1353
Figure 4
siRNA-ctrl
+
-
-
-
+
-
-
-
-
+
-
-
KDELR1-siRNA1
-
+
-
-
KDELR1-siRNA2
-
-
+
-
-
-
+
-
-
-
-
+
-
-
-
+
KDELR1-siRNA3
34kDa
Hs 819.T
KDELR1
26kDa
SW1353
15kDa
Cytosol
Nucleus
Cytosol
Nucleus
+
-
-
-
+
-
-
-
0.56 0.08 0.04 0.06
1.08 0.57 0.17 0.18
siRNA-ctrl
+
-
-
-
+
-
-
-
-
+
-
-
-
+
-
-
-
+
-
-
-
+
-
-
KDELR1-siRNA1
95kDa
-
-
+
-
-
-
+
-
KDELR1-siRNA2
-
-
+
-
-
-
+
-
-
-
-
+
-
-
-
+
70kDa
-
-
-
+
-
-
-
+
KDELR1-siRNA3
YAP1
55kDa
70kDa
YAP1
43kDa
0.32 0.66 0.75 0.72
0.65 0.76 0.84 0.97
55kDa
43kDa
0.97 0.43 0.28 0.41
0.35 0.81 0.99 0.79
0.10 0.65 0.66 0.82
- - - - -
95kDa
70kDa
55kDa
Tubulin
YAP1
Phos-tag
34kDa
26kDa
70kDa
15kDa
H2A
0.79 0.38 0.39 0.21
0.64 0.38 0.34 0.27
11kDa
95kDa
70kDa
Active-YAP1
55kDa
1.07 0.42 0.48 0.66
43kDa
1.24 0.52 0.65 0.48
55kDa
43kDa
Actin
34kDa

## Slide 3
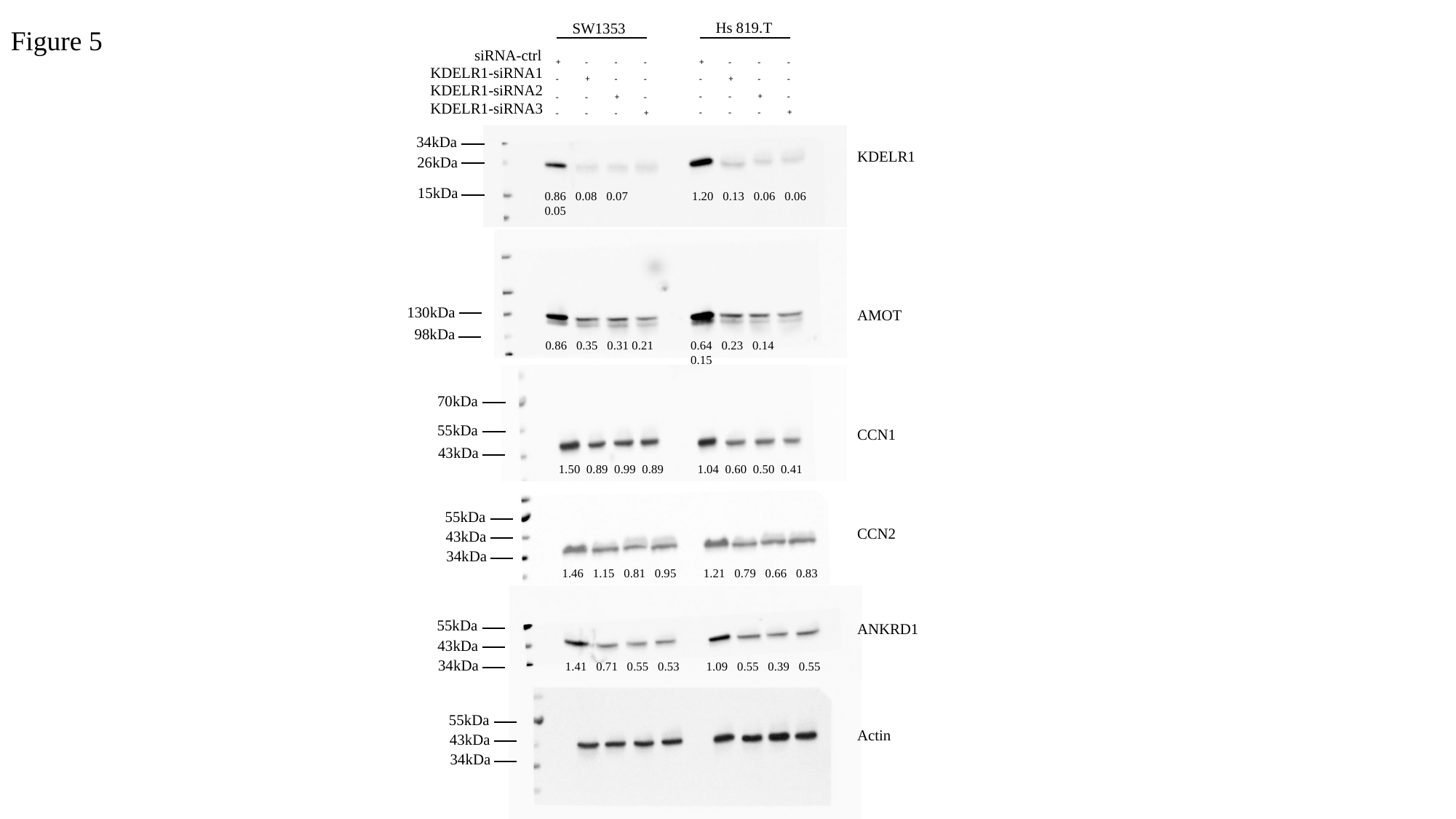

Hs 819.T
SW1353
Figure 5
siRNA-ctrl
+
-
-
-
+
-
-
-
-
+
-
-
-
+
-
-
KDELR1-siRNA1
-
-
+
-
KDELR1-siRNA2
-
-
+
-
-
-
-
+
-
-
-
+
KDELR1-siRNA3
34kDa
KDELR1
26kDa
15kDa
0.86 0.08 0.07 0.05
1.20 0.13 0.06 0.06
130kDa
AMOT
98kDa
0.86 0.35 0.31 0.21
0.64 0.23 0.14 0.15
70kDa
55kDa
CCN1
43kDa
1.50 0.89 0.99 0.89
1.04 0.60 0.50 0.41
55kDa
CCN2
43kDa
34kDa
1.46 1.15 0.81 0.95
1.21 0.79 0.66 0.83
55kDa
ANKRD1
43kDa
34kDa
1.41 0.71 0.55 0.53
1.09 0.55 0.39 0.55
55kDa
Actin
43kDa
34kDa

## Slide 4
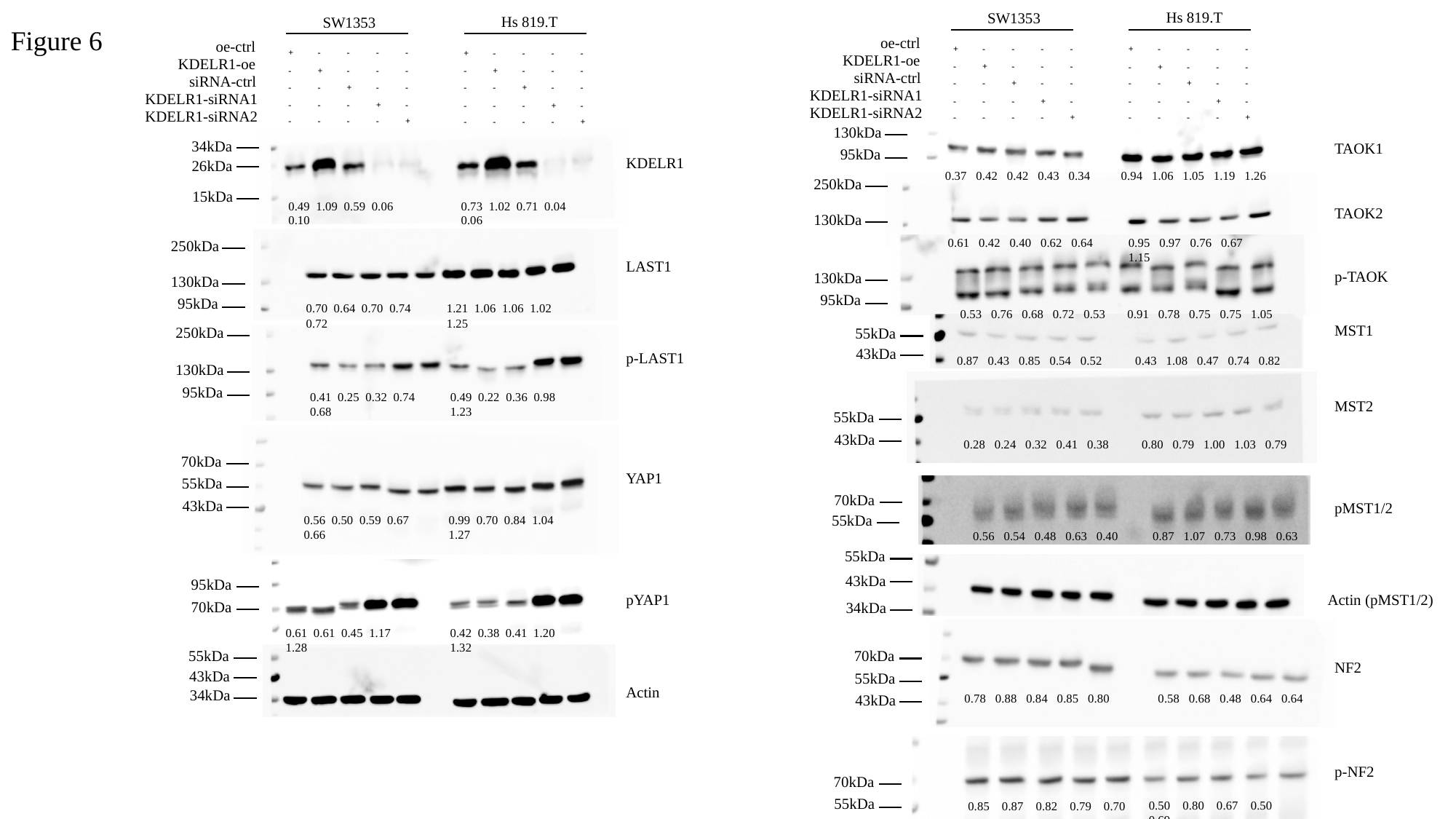

Hs 819.T
SW1353
Hs 819.T
SW1353
Figure 6
+
-
-
-
-
oe-ctrl
+
-
-
-
-
+
-
-
-
-
oe-ctrl
+
-
-
-
-
KDELR1-oe
-
+
-
-
-
-
+
-
-
-
KDELR1-oe
-
+
-
-
-
-
+
-
-
-
-
-
+
-
-
-
-
+
-
-
siRNA-ctrl
-
-
+
-
-
-
-
+
-
-
siRNA-ctrl
-
-
-
+
-
KDELR1-siRNA1
-
-
-
+
-
-
-
-
+
-
KDELR1-siRNA1
-
-
-
+
-
-
-
-
-
+
-
-
-
-
+
KDELR1-siRNA2
-
-
-
-
+
-
-
-
-
+
KDELR1-siRNA2
130kDa
34kDa
TAOK1
95kDa
KDELR1
26kDa
0.37 0.42 0.42 0.43 0.34
0.94 1.06 1.05 1.19 1.26
250kDa
15kDa
0.49 1.09 0.59 0.06 0.10
0.73 1.02 0.71 0.04 0.06
TAOK2
130kDa
0.61 0.42 0.40 0.62 0.64
0.95 0.97 0.76 0.67 1.15
250kDa
LAST1
p-TAOK
130kDa
130kDa
95kDa
95kDa
0.70 0.64 0.70 0.74 0.72
1.21 1.06 1.06 1.02 1.25
0.53 0.76 0.68 0.72 0.53
0.91 0.78 0.75 0.75 1.05
MST1
250kDa
55kDa
43kDa
p-LAST1
0.87 0.43 0.85 0.54 0.52
0.43 1.08 0.47 0.74 0.82
130kDa
95kDa
0.41 0.25 0.32 0.74 0.68
0.49 0.22 0.36 0.98 1.23
MST2
55kDa
43kDa
0.28 0.24 0.32 0.41 0.38
0.80 0.79 1.00 1.03 0.79
70kDa
YAP1
55kDa
70kDa
43kDa
pMST1/2
55kDa
0.56 0.50 0.59 0.67 0.66
0.99 0.70 0.84 1.04 1.27
0.56 0.54 0.48 0.63 0.40
0.87 1.07 0.73 0.98 0.63
55kDa
43kDa
95kDa
Actin (pMST1/2)
pYAP1
70kDa
34kDa
0.61 0.61 0.45 1.17 1.28
0.42 0.38 0.41 1.20 1.32
55kDa
70kDa
NF2
43kDa
55kDa
Actin
34kDa
43kDa
0.78 0.88 0.84 0.85 0.80
0.58 0.68 0.48 0.64 0.64
p-NF2
70kDa
55kDa
0.50 0.80 0.67 0.50 0.69
0.85 0.87 0.82 0.79 0.70
55kDa
43kDa
Actin
34kDa

## Slide 5
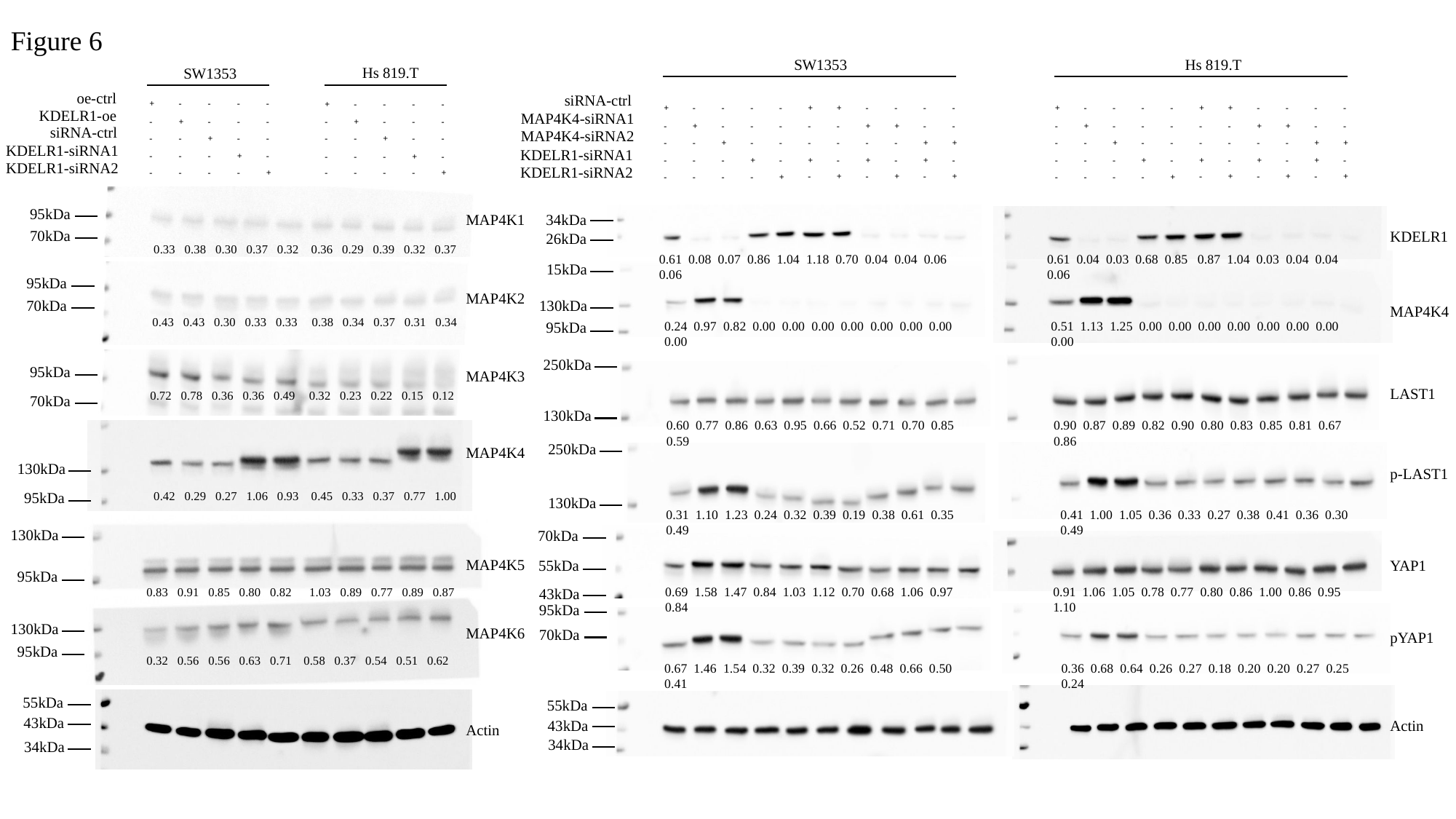

Figure 6
SW1353
Hs 819.T
Hs 819.T
SW1353
+
-
-
-
-
oe-ctrl
+
-
-
-
-
siRNA-ctrl
+
+
-
-
-
-
+
+
-
-
-
-
+
-
-
-
-
+
-
-
-
-
KDELR1-oe
-
+
-
-
-
-
+
-
-
-
MAP4K4-siRNA1
-
-
+
+
-
-
-
-
+
+
-
-
-
+
-
-
-
-
+
-
-
-
-
-
+
-
-
-
-
+
-
-
siRNA-ctrl
MAP4K4-siRNA2
-
-
-
-
+
+
-
-
-
-
+
+
-
-
+
-
-
-
-
+
-
-
-
-
-
+
-
KDELR1-siRNA1
-
-
-
+
-
+
-
+
-
+
-
+
-
+
-
+
-
-
-
-
+
-
-
-
-
+
-
KDELR1-siRNA1
-
-
-
-
+
-
-
-
-
+
KDELR1-siRNA2
-
+
-
+
-
+
-
+
-
+
-
+
-
-
-
-
+
-
-
-
-
+
KDELR1-siRNA2
95kDa
34kDa
MAP4K1
70kDa
KDELR1
26kDa
0.33 0.38 0.30 0.37 0.32
0.36 0.29 0.39 0.32 0.37
0.61 0.08 0.07 0.86 1.04 1.18 0.70 0.04 0.04 0.06 0.06
0.61 0.04 0.03 0.68 0.85 0.87 1.04 0.03 0.04 0.04 0.06
15kDa
95kDa
MAP4K2
70kDa
130kDa
MAP4K4
0.43 0.43 0.30 0.33 0.33
0.38 0.34 0.37 0.31 0.34
0.24 0.97 0.82 0.00 0.00 0.00 0.00 0.00 0.00 0.00 0.00
0.51 1.13 1.25 0.00 0.00 0.00 0.00 0.00 0.00 0.00 0.00
95kDa
250kDa
95kDa
MAP4K3
LAST1
0.72 0.78 0.36 0.36 0.49
0.32 0.23 0.22 0.15 0.12
70kDa
130kDa
0.60 0.77 0.86 0.63 0.95 0.66 0.52 0.71 0.70 0.85 0.59
0.90 0.87 0.89 0.82 0.90 0.80 0.83 0.85 0.81 0.67 0.86
250kDa
MAP4K4
130kDa
p-LAST1
95kDa
0.42 0.29 0.27 1.06 0.93
0.45 0.33 0.37 0.77 1.00
130kDa
0.31 1.10 1.23 0.24 0.32 0.39 0.19 0.38 0.61 0.35 0.49
0.41 1.00 1.05 0.36 0.33 0.27 0.38 0.41 0.36 0.30 0.49
130kDa
70kDa
MAP4K5
YAP1
55kDa
95kDa
0.69 1.58 1.47 0.84 1.03 1.12 0.70 0.68 1.06 0.97 0.84
0.91 1.06 1.05 0.78 0.77 0.80 0.86 1.00 0.86 0.95 1.10
43kDa
0.83 0.91 0.85 0.80 0.82
1.03 0.89 0.77 0.89 0.87
95kDa
130kDa
MAP4K6
70kDa
pYAP1
95kDa
0.58 0.37 0.54 0.51 0.62
0.32 0.56 0.56 0.63 0.71
0.67 1.46 1.54 0.32 0.39 0.32 0.26 0.48 0.66 0.50 0.41
0.36 0.68 0.64 0.26 0.27 0.18 0.20 0.20 0.27 0.25 0.24
55kDa
55kDa
43kDa
43kDa
Actin
Actin
34kDa
34kDa

## Slide 6
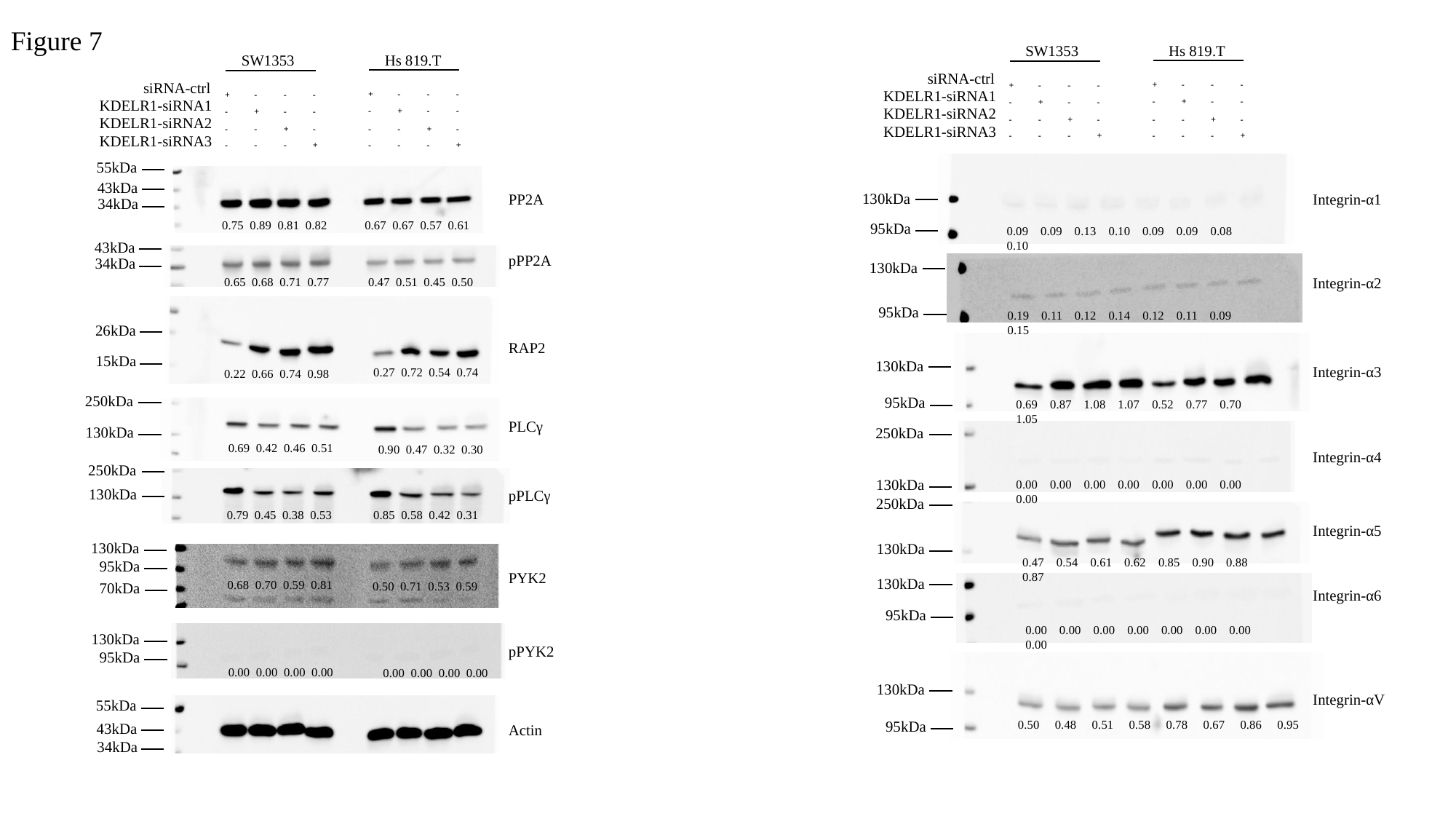

Figure 7
Hs 819.T
SW1353
Hs 819.T
SW1353
siRNA-ctrl
+
-
-
-
+
-
-
-
siRNA-ctrl
+
-
-
-
+
-
-
-
-
+
-
-
-
+
-
-
KDELR1-siRNA1
-
+
-
-
-
+
-
-
KDELR1-siRNA1
-
-
+
-
KDELR1-siRNA2
-
-
+
-
-
-
+
-
KDELR1-siRNA2
-
-
+
-
-
-
-
+
-
-
-
+
KDELR1-siRNA3
-
-
-
+
-
-
-
+
KDELR1-siRNA3
55kDa
43kDa
130kDa
PP2A
Integrin-α1
34kDa
0.75 0.89 0.81 0.82
0.67 0.67 0.57 0.61
95kDa
0.09 0.09 0.13 0.10 0.09 0.09 0.08 0.10
43kDa
pPP2A
34kDa
130kDa
Integrin-α2
0.65 0.68 0.71 0.77
0.47 0.51 0.45 0.50
95kDa
0.19 0.11 0.12 0.14 0.12 0.11 0.09 0.15
26kDa
RAP2
15kDa
130kDa
Integrin-α3
0.27 0.72 0.54 0.74
0.22 0.66 0.74 0.98
250kDa
95kDa
0.69 0.87 1.08 1.07 0.52 0.77 0.70 1.05
PLCγ
130kDa
250kDa
0.69 0.42 0.46 0.51
0.90 0.47 0.32 0.30
Integrin-α4
250kDa
130kDa
0.00 0.00 0.00 0.00 0.00 0.00 0.00 0.00
130kDa
pPLCγ
250kDa
0.79 0.45 0.38 0.53
0.85 0.58 0.42 0.31
Integrin-α5
130kDa
130kDa
0.47 0.54 0.61 0.62 0.85 0.90 0.88 0.87
95kDa
PYK2
130kDa
0.68 0.70 0.59 0.81
70kDa
0.50 0.71 0.53 0.59
Integrin-α6
95kDa
0.00 0.00 0.00 0.00 0.00 0.00 0.00 0.00
130kDa
pPYK2
95kDa
0.00 0.00 0.00 0.00
0.00 0.00 0.00 0.00
130kDa
Integrin-αV
55kDa
95kDa
0.50 0.48 0.51 0.58 0.78 0.67 0.86 0.95
43kDa
Actin
34kDa

## Slide 7
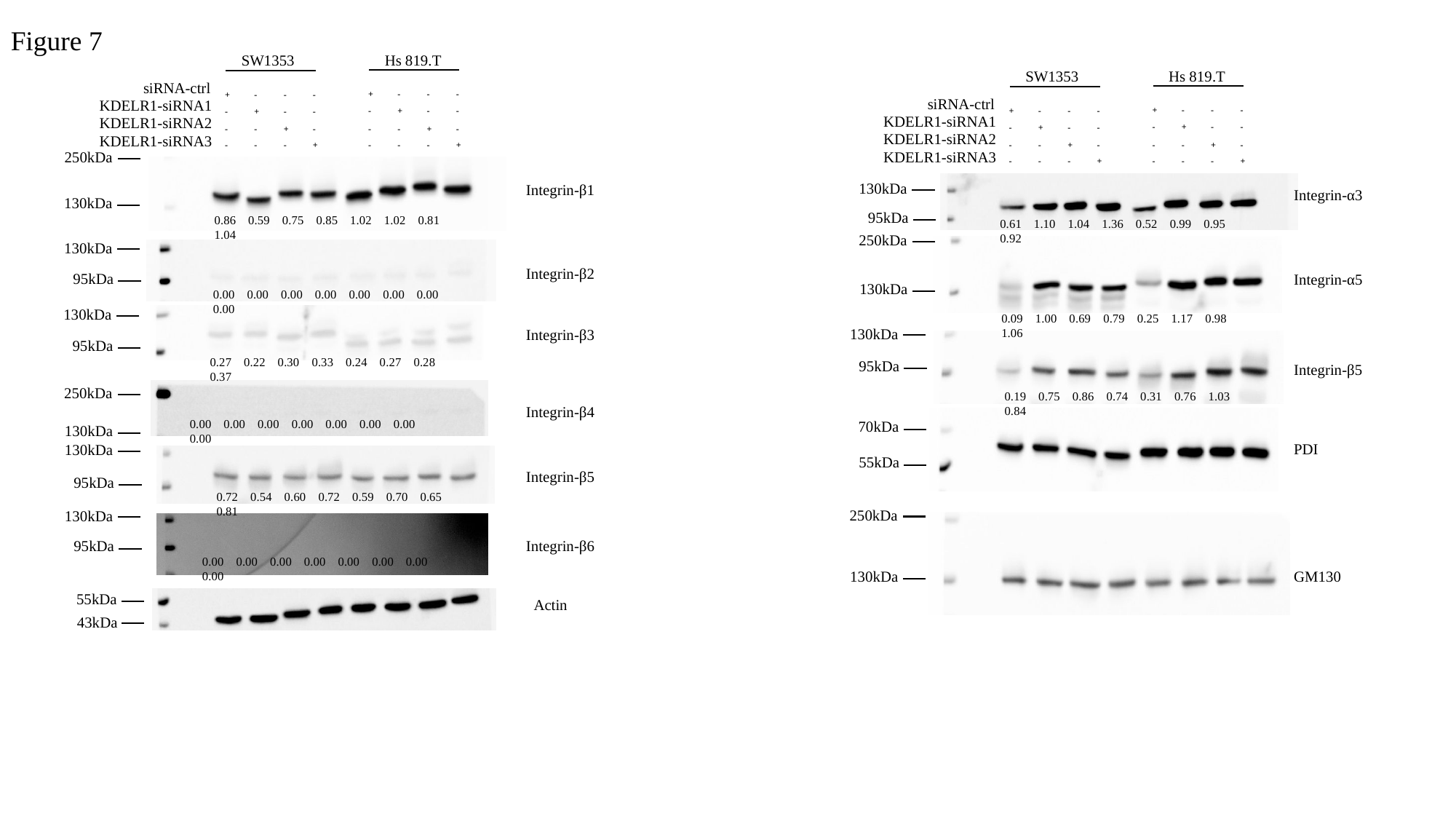

Figure 7
Hs 819.T
SW1353
Hs 819.T
SW1353
siRNA-ctrl
+
-
-
-
+
-
-
-
siRNA-ctrl
+
-
-
-
+
-
-
-
-
+
-
-
-
+
-
-
KDELR1-siRNA1
-
+
-
-
-
+
-
-
KDELR1-siRNA1
-
-
+
-
KDELR1-siRNA2
-
-
+
-
-
-
+
-
-
-
-
+
KDELR1-siRNA2
-
-
+
-
-
-
-
+
KDELR1-siRNA3
-
-
-
+
-
-
-
+
KDELR1-siRNA3
250kDa
130kDa
Integrin-β1
Integrin-α3
130kDa
95kDa
0.86 0.59 0.75 0.85 1.02 1.02 0.81 1.04
0.61 1.10 1.04 1.36 0.52 0.99 0.95 0.92
250kDa
130kDa
Integrin-β2
95kDa
Integrin-α5
130kDa
0.00 0.00 0.00 0.00 0.00 0.00 0.00 0.00
130kDa
0.09 1.00 0.69 0.79 0.25 1.17 0.98 1.06
130kDa
Integrin-β3
95kDa
0.27 0.22 0.30 0.33 0.24 0.27 0.28 0.37
95kDa
Integrin-β5
250kDa
0.19 0.75 0.86 0.74 0.31 0.76 1.03 0.84
Integrin-β4
0.00 0.00 0.00 0.00 0.00 0.00 0.00 0.00
70kDa
130kDa
PDI
130kDa
55kDa
Integrin-β5
95kDa
0.72 0.54 0.60 0.72 0.59 0.70 0.65 0.81
250kDa
130kDa
95kDa
Integrin-β6
0.00 0.00 0.00 0.00 0.00 0.00 0.00 0.00
130kDa
GM130
55kDa
Actin
43kDa

## Slide 8
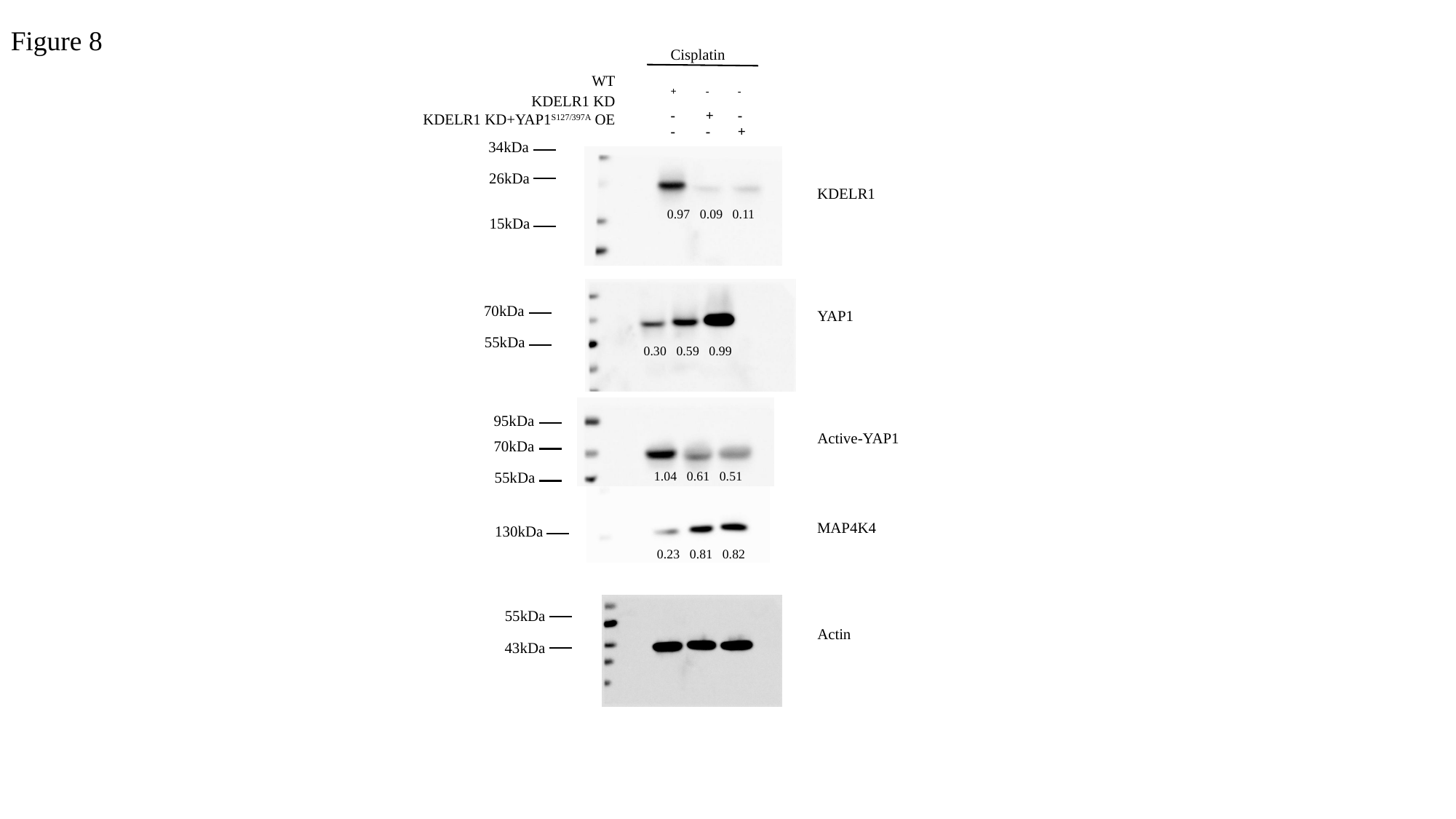

Figure 8
Cisplatin
WT
+
-
-
-
+
-
KDELR1 KD
-
-
+
KDELR1 KD+YAP1S127/397A OE
34kDa
26kDa
KDELR1
0.97 0.09 0.11
15kDa
70kDa
YAP1
55kDa
0.30 0.59 0.99
95kDa
Active-YAP1
70kDa
1.04 0.61 0.51
55kDa
MAP4K4
130kDa
0.23 0.81 0.82
55kDa
Actin
43kDa
